# Magnetic Correlations in Co/CoO Core/Shell Nanoparticles Decorated on Graphene: Insights from a Modified 2D Law of Approaching Magnetic Saturation

**DOI:** 10.3390/nano16191201

**Published:** 2026-09-23

**Authors:** Ekaterina S. Nazarenka, Aleksander L. Danilyuk, Nikolai G. Kovalchuk, Artjom O. Konakov, Julia A. Fedotova, Serghej L. Prischepa

**Affiliations:** 1 Department of Micro- and Nanoelectronics, Belarusian State University of Informatics and Radioelectronics, 220013 Minsk, Belarus; katyanaz2001@gmail.com (E.S.N.); danilyuk@bsuir.by (A.L.D.); 2 Laboratory “Integrated Micro- and Nanosystems”, Research and Development Department, Belarusian State University of Informatics and Radioelectronics, 220013 Minsk, Belarus; n.kovalchuk@bsuir.by; 3 Research Institute for Physical Chemical Problems, Belarusian State University, 220030 Minsk, Belarus; artjom7777@gmail.com; 4 Institute for Nuclear Problems, Belarusian State University, 220030 Minsk, Belarus; julia@hep.by; 5 Department of Information Security, Belarusian State University of Informatics and Radioelectronics, 220013 Minsk, Belarus

**Keywords:** Co/CoO core/shell nanoparticles, graphene, random anisotropy model, law of approaching magnetic saturation, exchange bias, magnetic correlations, spintronics, 2D magnetism

## Abstract

Graphene-based hybrid magnetic systems are promising platforms for spintronic applications, yet the mechanisms governing magnetic correlations in these two-dimensional (2D) architectures remain incompletely understood. Here, we investigate the magnetic behavior of Co/CoO core–shell nanoparticles electrochemically deposited on graphene, focusing on the 25–50 K range, where the coercivity and exchange bias field reach their maximum values. Using a modified 2D integral-law framework for approaching magnetic saturation, adapted for 2D systems via the Meyer integral transform, we reconstruct the real-space correlation functions (CFs) of the magnetic anisotropy axes and extract the main micromagnetic parameters. The analysis reveals three distinct types of CFs, monotonic, non-monotonic, and jump-like, which we tentatively associate with different microstructural configurations, including isolated nanoparticles, dense clusters, and sparse conglomerates separated by graphene domain boundaries. The monotonic CFs exhibit long-range coherence of the magnetic anisotropy axes extending over nearly one micrometer. We propose that this long-range behavior may arise from a dual-scale mechanism: indirect RKKY-type exchange through the graphene layer, combined with strong local anisotropy from the CoO shells, which pins the magnetization of individual Co cores via exchange bias. These interpretations are presented as plausible hypotheses, and further experimental verification is required.

## 1. Introduction

Graphene, a single two-dimensional (2D) sheet of carbon atoms arranged in a honeycomb lattice, has attracted considerable attention because of its exceptional carrier mobility, mechanical strength, and high specific surface area [1]. In spintronics, technologies that exploit the electron spin in addition to charge, graphene provides a promising medium for information transport [2]. However, pristine graphene is intrinsically diamagnetic because its sp^2^-hybridized carbon network contains paired electrons forming σ- and π-bonds, resulting in no net magnetic moment [2,3]. Inducing and controlling magnetic order in graphene therefore remains a major challenge in condensed matter physics and materials science, with potential applications ranging from spin filters and nonvolatile memory devices to biomedical platforms for targeted therapy and diagnostics [2,3,4].

Theoretical and experimental studies have demonstrated that magnetism in graphene can emerge through various strategies that disrupt its symmetric bond configuration, including the introduction of point or line defects, edge modification of nanoribbons and nanoflakes, sp^3^-functionalization, interlayer rotation (as in magic-angle twisted bilayer graphene), and heteroatom doping [2,3,5,6]. These approaches generate unpaired electrons and localized states that can interact to form magnetic clusters, thereby imparting magnetic properties to the initially non-magnetic carbon lattice [3,5]. In the context of spintronics, the spin degrees of freedom and their controlled manipulation are of paramount importance. In pure graphene, the spin–orbit interaction is extremely weak, resulting in relatively stable spin states and long spin-relaxation times. However, defects, impurities, and substrate interactions can enhance spin–orbit coupling [7,8], thereby enabling spin-dependent phenomena [7,9]. Such modifications may nevertheless compromise the structural integrity and charge-transport properties of the host material.

To overcome these limitations, decorating graphene with magnetic nanoparticles (NPs) has emerged as a promising hybrid strategy. This approach combines the robust magnetic moments of transition metals with the superior conductivity of graphene, enabling the development of tunable, multifunctional nanocomposites [2,10,11,12]. The interplay between magnetic NPs and the graphene host can also give rise to novel phenomena such as spin-filtering effects, negative differential resistance, and magnetoresistance variations, offering pathways to advanced nanoelectronics and spintronic devices [10,11,13].

Several experimental studies have developed various methods for decorating graphene with magnetic NPs. For instance, graphene decorated with barium ferrite NPs exhibits soft ferrimagnetic behavior with coercivity and saturation magnetization significantly altered relative to the pure ferrite phase; spin disorder at the graphene/NP interface plays a critical role in modifying the magnetic response [4]. Similarly, α-Fe_2_O_3_-decorated graphene demonstrates strong interparticle interactions and solvent-dependent superparamagnetic blocking behavior, highlighting the sensitivity of such hybrid systems to synthesis conditions [10]. In Ref. [14], a nanohybrid structure was developed to achieve room-temperature ferromagnetism using a simple, efficient, and reproducible solvothermal synthesis method. In that system, the growth of Co NPs on the surface of reduced graphene oxide nanosheets switches the magnetic interactions from superparamagnetic to ferromagnetic (FM) at room temperature.

The fabrication of FM NPs with a Co core and an antiferromagnetic (AFM) CoO shell, with an average size of about 40 nm, was reported using electrochemical deposition on copper and copper-covered graphene substrates [12]. Core/shell architectures are particularly compelling: the interfacial exchange between the FM Co core and the AFM CoO shell provides a significant exchange bias, which can stabilize magnetic order and overcome the superparamagnetic limit even at reduced dimensions. Measurements of magnetic hysteresis loops *M*(*H*) at different temperatures down to *T* = 4 K revealed a pronounced exchange bias *H_eb_* and an unusual temperature dependence of both the coercivity *H_c_* and *H_eb_* [12]. In Ref. [15], these results were analyzed using a correlation function approach for the magnetic anisotropy axes. However, the authors presented results only for test samples in which Co NPs were deposited on copper, and they demonstrated that analyzing hysteresis loops over a wide temperature range is a complex task. Notably, a conductive substrate significantly influences the magnetic interaction between particles, and the interaction mechanism changes with temperature. Moreover, the magnetic properties of NPs on a copper substrate and on conductive graphene differ substantially, as demonstrated in [12].

A theoretical gap persists in understanding how magnetic correlations propagate across a 2D graphene sheet. Traditional analysis often relies on the Random Anisotropy Model (RAM), which assumes a 3D bulk environment and a rapid exponential decay of magnetic correlations. On a 2D graphene substrate, however, the conducting medium can host indirect Ruderman–Kittel–Kasuya–Yosida (RKKY)-type exchange interactions that are markedly more persistent [16]. Recent experimental and theoretical studies have demonstrated that graphene can mediate long-range magnetic interactions, with coupling between hydrogen adatom magnetic moments extending over distances exceeding 10 nm [17], and that extended defects or impurity lines can significantly enhance the range of such interactions [16]. Furthermore, RKKY-like exchange coupling has been observed between Fe adatoms on extended graphene [18], and gate-tunable magnetism of C adatoms on graphene has been analyzed via the RKKY model [19], providing a solid foundation for interpreting substrate-mediated magnetic correlations in graphene-based hybrids. While Co/CoO NP decoration of graphene has been studied in the context of magnetotransport [11,13], the role of the underlying graphene domain boundaries in modulating the spatial distribution and magnetic correlation of such NPs has not been systematically explored.

This work addresses this gap by applying a modified 2D Law of Approaching Magnetic Saturation (LAMS) framework to a well-characterized Co/CoO/Gr system [12]. The present study does not report new sample synthesis or structural characterization. The experimental data summarized in Section 2.1, including CVD graphene growth, electrochemical deposition, Scanning Electron Microscopy (SEM) (Hitachi S4800), X-ray Photoemission Spectroscopy (XPS) (ESCALAB 250Xi), High-Resolution Transmission Electron Microscopy (HRTEM) (FEI Titan 80–300) analysis, and basic magnetization curves, have been reported previously in Ref. [12]. We move beyond the limitations of the canonical RAM [20] by using an integral version of the LAMS, adapted for 2D systems via the Meyer integral transform [21,22]. By analyzing magnetization data at low temperatures (below 100 K), we reconstruct the correlation function (CF) *C*(*z*) of the magnetic anisotropy axes in real space. Our findings reveal that the interplay between local shell-driven anisotropy and long-range, graphene-mediated exchange interactions creates a unique magnetic landscape with unexpectedly large correlation lengths. Furthermore, we identify three distinct types of CFs, monotonic, non-monotonic, and jump-like, each corresponding to different microstructural configurations (isolated NPs, dense clusters, and sparse conglomerates separated by graphene domain boundaries). This classification provides a comprehensive picture of the magnetic interactions in 2D hybrid systems and offers a quantitative framework for understanding and potentially controlling nanoscale magnetic order on the micrometer scale, which could be relevant for future spintronic architectures.

It is important to clarify which elements of the mathematical framework employed here are newly derived and which were previously established. The general integral formulation of the 2D LAMS was originally proposed by Chudnovsky [22] and was subsequently modified into the Meyer integral transform and applied to magnetic NP systems in our earlier works [15,21]. In the present study, we extend this formalism to the Co/CoO/Gr system by employing new expressions for the image function (Equation (3)) and the correlation function (Equation (4)), specifically adapted to the core/shell morphology and the graphene-mediated interactions in this hybrid material. The application of this framework to Co/CoO NPs on graphene, together with the identification of three distinct correlation regimes (monotonic, non-monotonic, and jump-like), constitutes the new contribution of this work.

## 2. Materials and Methods

### 2.1. Experimental Data Summary

Since the main stages of Co NP formation on graphene and their characterization have been described in detail in [12], they are briefly summarized here. This summary serves as the experimental foundation for our theoretical analysis; no new synthesis or structural characterization is reported in this work. Graphene was synthesized by atmospheric-pressure chemical vapor deposition (CVD) on previously cleaned Cu foil (Alfa Aesar 99.8% purity, NY), using n-decane (C_10_H_22_) as the precursor (Condor Oil) and nitrogen (Promgaz) as the carrier gas. The synthesis temperature was 1050 °C, with N_2_ and H_2_ flow rates of 100 cm^3^/min and 6 cm^3^/min, respectively, and the C_10_H_22_ feed rate of 4 µL/min [23].

Co NPs were subsequently electrochemically deposited on graphene supported on copper foil at room temperature under constant current conditions. The structural parameters of the Co/CoO/Gr system, as determined from SEM, XPS, HRTEM, and Raman spectroscopy (Confotec NR 500), are summarized as follows. SEM imaging revealed that the electrodeposited NPs have diameters of approximately 100 nm, with a noticeable size distribution characteristic of electrochemical deposition on graphene [12]. XPS analysis confirmed the presence of both metallic Co and an oxide phase identified as CoO, while HRTEM imaging directly confirmed the core–shell structure [12] and provided information on the thicknesses of the Co core, *d*_Co_, and the CoO shell, *d*_CoO_, with *d*_Co_/*d*_CoO_ ≈ 0.5. Accordingly, the NPs consist of an FM Co core and an AFM CoO shell, and the exchange bias arises from the interfacial exchange interaction between the FM core and the surrounding AFM shell [24]. The Co core size of diameter of 20 nm is consistent with a single-domain structure [25]. The areal density of the NPs was not determined due to the heterogeneous spatial distribution resulting from preferential nucleation at graphene defects and domain boundaries (see discussion in Section 3.2.3). From Raman spectroscopy, the average graphene domain size was estimated to be in the range of 40–90 nm (see Supplementary Information, Figure S1).

Magnetization (*M*) versus applied magnetic field (*H*) measurements, performed using a Vibrating Sample Magnetometer, revealed hysteresis loops over the entire studied temperature range, from 4 K up to 300 K. The hysteresis loops were recorded after field-cooling to 4 K in the presence of a +10 kOe magnetic field applied parallel to the sample surface. More detailed information on the sample characteristics and experimental results is given elsewhere [12].

Figure 1 shows the magnetization hysteresis loops, *M*(*H*), measured at *T* = 25 and 50 K for the Co/CoO NPs on graphene. The *M*(*H*) curves at *T* = 4 and 300 K were reported in Ref. [12]. Table 1 summarizes the corresponding exchange bias field, *H_eb_*, saturation magnetization, *M_S_*, and coercivity, *H_c_*.

As shown in Table 1, *H_c_* and *H_eb_* exhibit a nonmonotonic temperature dependence, with a maximum in the 25–50 K range. This behavior suggests a strong influence of the CoO shell, particularly its thickness, and of the CoO/Co interface on magnetic ordering [26,27,28]. Exchange bias and coercivity in core/shell nanostructures are of considerable interest because they help clarify the underlying mechanisms and enable practical applications [28,29,30,31,32,33,34]. Therefore, we focus below on the 25–50 K temperature range, where *H_c_* and *H_eb_* reach their maximum values.

From the reported *H_eb_* values in Table 1, it follows that the exchange bias blocking temperature $T_{B,EB}$ is between 200 and 300 K.

### 2.2. Mathematical Framework: Modified 2D LAMS

RAM is commonly used to analyze and interpret magnetic measurement results for this type of magnetic composite. However, its applicability is limited by underlying approximations, specifically by the choice of the correlation function C(z) of the magnetic anisotropy axes in real space, which describes the distance over which these axes are correlated. In the canonical RAM, these correlations are represented exclusively by an exponential function, C(z)~exp(−z), where $z=r/R_{NP}$ is the coordinate normalized by the radius of the metallic Co core, $R_{NP}\approx 10$ nm, as determined from HRTEM analysis [12]. In addition, rigid relationships are imposed between the external magnetic field and the exchange field. As a result, this model reproduces the experimental data on the approach to saturation only in limiting cases *H* << *H_ex_* and *H* >> *H_ex_*.

An important intermediate regime occurs when the magnetic field is comparable to the exchange interaction field. This regime requires modifying the RAM to account for sample dimensionality [22] and the impact of the conducting host material, which couples the FM NPs via indirect exchange interactions [16,18,35]. All these factors directly affect the CF form. For example, the behavior of ferromagnetic NPs embedded in carbon nanotubes is described by oscillating long-range correlations; this reflects the influence of the conduction electron spins of the inner walls on the correlations of the NPs’ magnetic anisotropy axes [21,36].

A distinctive feature of the system under study is its 2D nature. In this case, the LAMS undergoes additional modifications that should be taken into account. Before elaborating the 2D formulation, it is important to clarify the physical justification for treating the Co/CoO NP array on graphene as an effectively two-dimensional magnetic system. While the individual NPs possess a 3D core/shell morphology with finite height, their spatial distribution is confined to the graphene surface. In this sense, the system can be considered two-dimensional with respect to the arrangement of NP centers.

The applicability of the 2D LAMS to such systems has been previously demonstrated for NP arrays on conducting supports [21]. The key condition for the validity of the 2D approximation is that the thickness of the NP layer ($t_{NP}$) be small compared to the magnetic correlation length ($L_{corr}$) over which the anisotropy axes are correlated. In our case, $t_{NP}\approx 20$–100 nm (based on the NP diameter [12]), while the reconstructed correlation lengths extend up to nearly 1 μm (see Section 3.3). Thus, $t_{NP}/L_{corr}≲0.1$, which comfortably satisfies the 2D criterion. Moreover, the 2D formulation of the LAMS has been successfully applied to other quasi-2D systems, such as nanogranular thin films [22,37]. Although the individual magnetic entities in these systems are three-dimensional, their confinement to a plane and the planar geometry of the exchange interactions justify using a 2D model. We therefore consider the 2D LAMS framework adopted here to be physically appropriate for the Co/CoO/Gr system, while acknowledging that the finite NP height may introduce minor quantitative corrections beyond the scope of the present analysis.

The initial refinement of the RAM, proposed in Ref. [22], describes the LAMS in integral form. This formulation enables the direct determination of the CFs of the random anisotropy axes from the image functions that define the law, thereby bypassing the limitations of the canonical RAM [20,37,38,39]. Specifically, the LAMS for 2D magnetic systems corresponds to the Meyer integral transform [21,40]. Using this transform, one can determine the CF, as well as the exchange and random anisotropy fields. Thus, the LAMS for a 2D ferromagnet, obtained in Refs. [15,21], can be expressed as

(1) $$F(p)=\frac{32p^{3/2}}{2\pi }{\left(\frac{H_{ex}}{H_{a}}\right)}^{2}\frac{\delta M(p)}{M_{S}}=\int_{0}^{\infty }z^{\frac{3}{2}}C\left(z\right)\sqrt{pz}K_{1}\left(pz\right)dz,$$

where $\delta M\left(p\right)=M_{S}-M(H)$, $K_{1}$ is the modified Bessel function of the second kind of the first order, $p={\left(H/H_{ex}\right)}^{1/2}$, and M(H) is the magnetization in the region of the LAMS. From integral Equation (1), it follows that $C\left(z\right)$ is unambiguously determined by the image function *F*(*p*) and must be updated as *F*(*p*) changes.

From Equation (1), it also follows that, based on the experimental *M*(*H*) data, the following “experimental” image function can be obtained:

(2) $$F_{exp}\left(p\right)=kp^{3/2}\frac{\delta M(p)}{M_{S}},$$

where *k* = (32/2π)(*H_ex_*/*H_a_*)^2^ will be used as a normalization coefficient to bring into correspondence experimental and calculated data to obtain the relation (*H_ex_*/*H_a_*), see below.

The results extracted from the experimental data $F_{exp}$ versus p dependencies for different $H_{ex}$ values and two temperatures are shown in Figure 2 (*T* = 50 K) and Figure 3 (*T* = 25 K) by blue symbols. The range of exchange fields ($H_{ex}=800–2400$ Oe) was selected based on the RAM framework applied to similar magnetic NP systems. Within this approach, the ensemble-averaged exchange field is $H_{ex}=2A/M_{s}R_{NP}^{2}$, and the random anisotropy field is $H_{a}=2K/M_{s}$, where K is the effective anisotropy constant related to $H_{c}$ [22]. To justify this range, we refer to experimental studies on Co/CoO core/shell nanostructures [33] and Co-decorated graphene nanohybrids [14,41], which provide $H_{c}$ values for analogous systems. The lower limit ($H_{ex}=500$ Oe) is set because below this value, the extracted $R_{NP}$ increases beyond our experimental data, and $H_{a}$ grows unrealistically. The upper limit ($H_{ex}<3$ kOe) is constrained by the external field range (7–10 kOe); above 3 kOe, $H_{a}$ decreases below $H_{ex}$, leading to a coercivity inconsistent with experiment.

As can be seen from Figure 2 and Figure 3, varying *H_ex_* has a significant impact on the value of *F_exp_*(*p*). It follows that $F_{exp}$ versus p exhibits a non-monotonic behavior and possesses a distinct maximum. As *p* increases, the LAMS region typically falls on the descending portion of the *F*(*p*) curve. Consequently, the CF was investigated within the specific magnetic field range where *F*(*p*) characterizes the LAMS. Initial estimates indicate that the experimental data are well approximated by the analytical relationships defined by the function [40]

(3) $$F_{anl}(p)=k{\left(2\pi \right)}^{-1/2}a^{1-\mu }p^{\mu }K_{1}\left(ap/2\right)K_{\mu -1/2}\left(ap/2\right),$$

where a and μ are model parameters, $K_{\mu -1/2}$ is the modified Bessel function of the second kind of the μ − 1 order.

In this case, according to the Meyer integral transform, the correlation function for the image function described by Equation (3) is *C*(*z*) = 0 at 0 < *z* < *a* and

(4) $$C(z)=z^{-\mu -\frac{3}{2}}{\left(z^{2}-a^{2}\right)}^{-\frac{\mu }{2}}P_{\frac{1}{2}}^{\mu }(z/a),$$

at *z* > *a*. Here $P_{\frac{1}{2}}^{\mu }(z/a)$ is the spherical (associated Legendre) function. Spherical function provided that [42]

$$\left|\frac{z/a-1}{z/a+1}\right|<1,$$

is determined as

(5) P12μza=1Γ(1−μ)z−1z+1−μ/2z+12−1F12−1/2;−1/2−μ;1−μ;z/a−1z/a+1,

where Γ(1−μ) is the gamma function, and F12(za, μ) is the hypergeometric function.

The results of calculating the analytical image function *F_anl_*(*p*) from Equation (3) with the parameter *a* = 1.0 and the exchange field *H_ex_* from 800 to 1800 Oe are shown by the red lines in Figure 2 and Figure 3. In this case, the function *F_anl_*(*p*) turns out to be more sensitive to changes in *H_ex_*, and varying *µ* in the range from −0.8 to −0.6 has very little effect on its value. Note that to bring the curves *F_exp_*(*p*) and *F_anl_*(*p*) into correspondence, fitting was used using the normalization parameter *k*, which depends on the field ratio (*H_ex_*/*H_a_*) and is independent of *µ*.

The corresponding CFs, determined using the inverse Meyer transform and describing the experimental data through the appropriate image function, are shown in Figure 4.

The parametric analysis reveals a clear functional separation: whereas the exchange field *H_ex_* governs the shape of the analytical image function *F_anl_*(*p*), the parameter *μ* determines the character of the correlation function *C*(*z*). As illustrated in Figure 4, varying *μ* (at fixed *a*) induces a profound transformation in *C*(*z*). The correlation functions can assume qualitatively distinct forms: monotonic, non-monotonic with a single maximum, or discontinuous with abrupt amplitude jumps at specific distances *z*. Remarkably, these diverse regimes emerge from relatively small variations in *μ*, highlighting the critical role of this parameter in shaping the magnetic correlation landscape.

Over a narrow range of *µ*, as *µ* increases from −0.98 to −0.6, *C*(*z*) changes drastically. It shifts from a monotonic increase (Figure 4e,f), which approaches saturation (Figure 4c,d), to non-monotonic behavior. This latter behavior is characterized by a maximum at *z* = 1.7–3.1 (Figure 4a,b), followed by a decrease with further increase in *z*. In general, an increase in the absolute value of *µ* leads to an increase in the amplitude of the CF, its saturation, and a subsequent further increase. Additionally, for certain critical values of the parameter *µ* = *µ_c_*, within the range shown in Figure 4, the correlation function *C*(*z*) exhibits discontinuities or jumps at distances of approximately $7R_{NP}\approx 70$ nm, where $R_{NP}=10$ nm is the Co core radius (Figure 4a–f). This range is limited by the *µ* values that correspond to a smooth *C*(*z*).

In Figure 5, we show the calculated *C*(*µ*) dependencies for two distinct *z* values—one corresponding to a smooth CF and the other to a regime in which jumps appear. As seen in this figure, the CF amplitude decreases as *µ* increases. Furthermore, the difference between the CFs for $\mu =\mu_{c}$ and $\mu \neq \mu_{c}$ becomes smaller. The presence of such a layered periodicity in the *C*(*µ*) dependence for $z>z_{c}$ indicates a strong inhomogeneity of the local exchange and anisotropy fields characterized by the parameter *µ*.

The various *C*(*z*) dependencies obtained indicate that, even for similar LAMSes, the physics of magnetic interactions between Co/CoO NPs on graphene is rich and diverse.

The parameter μ is a model parameter entering the analytical image function (Equation (3)) and, through the inverse Meyer transform, determines the shape of the correlation function C(z) (Equation (4)). Its influence on $F_{\mathrm{anl}}(p)$ is substantially weaker than that of $H_{\mathrm{ex}}$ and a, because the integral transform effectively averages over the μ-dependence of C(z). Consequently, the extracted micromagnetic parameters exhibit only a weak sensitivity to μ (variations of no more than 2–3%). In contrast, μ strongly affects the shape of C(z), controlling the position of the maximum and the character of the decay (or jump) in non-monotonic correlation functions. We therefore suggest that μ is physically related to the degree of inhomogeneity of the local exchange and anisotropy fields in the nanoparticle array. A more detailed quantitative correlation between μ and specific microstructural parameters (particle density, cluster size, interparticle coupling) requires further investigation and is beyond the scope of this work.

### 2.3. Model Evaluation and Parameter Extraction

As a first step toward clarifying the mechanisms of magnetic interaction between Co/CoO NPs on graphene, we estimate the random anisotropy field *H_a_* based on the obtained CF. Because we have already defined the range of possible exchange field values, each specific *H_ex_* directly corresponds to its own value of *H_a_*. Furthermore, by substituting the obtained CFs into Equation (1) alongside the derived values of *H_ex_* and *µ*, we reasonably reproduce the experimental *M*(*H*) dependence.

The fitting procedure used to determine the model parameters $H_{\mathrm{ex}}$, $H_{a}$, μ, and a proceeds as follows. First, an appropriate analytical image function $F_{\mathrm{anl}}(p)$ is selected from the Meyer integral transform tables (Equation (3)), and the exchange field $H_{\mathrm{ex}}$ is fixed. The normalization parameter k in Equation (2), which depends on the ratio $H_{\mathrm{ex}}/H_{a}$, is then varied to bring $F_{\exp}(p)$ and $F_{\mathrm{anl}}(p)$ into correspondence, yielding an acceptable range for μ and a. At fixed a=1, the allowed range of μ was found to be from −1 to −0.5.

The uniqueness of the procedure is determined by the choice of the image function (Equation (3)) and by the fixed value of $H_{\mathrm{ex}}$. The procedure is sensitive to the choice of $H_{\mathrm{ex}}$ and a. Strong correlations exist between $H_{\mathrm{ex}}$ and $H_{a}$ (through k) and between $H_{\mathrm{ex}}$ and a. However, since a is fixed at 1, the latter correlation does not affect the analysis. The correlation between $H_{\mathrm{ex}}$ and μ is very weak. The extracted micromagnetic parameters vary by no more than 2–3% with changes in μ. The estimated uncertainty of the extracted parameters is 5–15%, depending on the scatter of the experimental data.

Figure 6 and Figure 7 compare the experimental and theoretical results, showing the measured *δM(H)*/*M_S_* dependence (symbols) alongside the calculated curves (solid lines) obtained via the integral transformation in Equation (1) and the derived CF, Equation (4). Good agreement between *δM*(*H*)/*M_S_* and *F*(*p*) is observed at *H* > 2.744–2.89 kOe (*T* = 50 K) and *H* > 2.46–2.89 kOe (*T* = 25 K), which falls well within the validity range of the LAMS model.

The quality of the fit was quantified by calculating the root-mean-square error (RMSE) between the experimental and calculated dependencies $\delta M(p)/M_{s}$. The RMSE values range from 10^−5^ to 10^−1^ for all considered parameter sets, confirming good agreement between theory and experiment. The RMSE as a function of p for different $H_{ex}$ values are presented in the Supplementary Information (Figure S2a,b).

The LAMS region for our system was identified as 3–10 kOe (see Figure 1). This interval was used consistently in the fitting procedure, and the portions of the measured field range where $F_{\mathrm{anl}}(p)$ and $F_{\exp}(p)$ deviate from each other were excluded from the analysis. An additional check showed that the contribution of fields below 3 kOe may introduce a systematic uncertainty not exceeding 3–5%. Given the noticeable discrepancy between the experimental and analytical image functions in this low-field region, this contribution was neglected.

Figure 8 shows the calculated anisotropy fields as a function of the exchange field. As *H_ex_* increases from 0.6 to 2.4 kOe, the $H_{a}$ decreases from 6.0 to 4.0 kOe at *T* = 25 K and from 5.5 to 3.9 kOe at *T* = 50 K, exhibiting a clear tendency toward $H_{a}$ saturation when *H_ex_* > 1.8 kOe. Notably, variations in the *µ* parameter result in a change in the $H_{a}$ field of no more than 2–3%.

The *δM*(*H*)/*M_S_* dependencies were also calculated for various *H_ex_* (and their corresponding $H_{a}$), as shown in Figure 9. In this calculation, the relationship between *H_ex_* and $H_{a}$ was determined from the dependencies plotted in Figure 8. As can be seen, within the LAMS region, the *δM*(*H*)/*M_S_* dependencies are virtually identical for all *H_ex_*.

The calculations reveal that the random anisotropy field lies within the range of 3.9–6.0 kOe, depending on the exchange field, which was varied from 0.6 to 2.4 kOe. The obtained *H_a_*(*H_ex_*) dependences, described by Equation (1) within the framework of the LAMS, primarily establish a relationship between the random anisotropy and exchange fields.

To refine these values and ensure consistency with experimental data, we perform additional estimations of key magnetic parameters using the modified RAM. These estimations incorporate results derived from the integral LAMS model. The literature values for Co NPs were adopted for several parameters, including the commonly reported saturation magnetization *M_S_* = (1.1–1.4) × 10^6^ A/m [43], exchange constant *A* = (1.3–2.7) × 10^−11^ J/m [43,44], and uniaxial anisotropy constant *K* = (2.1–3.9) × 10^5^ J/m^3^ [45]. The governing equations of the RAM can be reduced to straightforward relations between micromagnetic parameters [15,36,39]. Furthermore, the interfacial exchange energy per unit area, $J_{\mathrm{eb}}=H_{eb}M_{S}t_{FM}$, where $t_{FM}$ is the effective ferromagnet thickness (in our case, 2*R_NP_*), is estimated using the Meiklejohn–Bean model [46,47,48].

By solving the RAM equation system with input parameters *H_a_*, *H_ex_*, *M_S_*, *H_c_*, and *A*, we determine the *T_B_*, *R_NP_*, and *K* values. The resulting parameters are summarized in Table 2. Notably, the magnetic parameters derived from the LAMS model for the Co/CoO NP array on graphene show good agreement with the data usually reported in the literature. Moreover, the high blocking temperatures obtained confirm that the NPs remain in a ferromagnetic state at the measurement temperatures of 25 and 50 K.

Based on the estimated parameters, the following values were selected for further discussion: *H_ex_* = 0.6–1.0 kOe, *H_a_* = 4.8–6.0 kOe, and *K* = (3.0–3.6) × 10^5^ J/m^3^, and *R_NP_* = 10 nm.

Before proceeding to the discussion, we would like to clarify the terminology related to the blocking temperatures reported in this work. In the following, we distinguish between two physically distinct quantities. The first is the superparamagnetic blocking temperature, denoted as $T_{B,SP}$, which refers to the temperature below which the magnetic moment of a single-domain NP is blocked along its easy axis due to the anisotropy energy barrier. The second is the exchange-bias blocking temperature, denoted as $T_{B,EB}$, which is the temperature above which the exchange bias field $H_{eb}$ vanishes because the antiferromagnetic pinning (here, the CoO shell) loses its stability. These two temperatures have different microscopic origins and are not necessarily equal. In Table 2 and throughout the text, the values of $T_{B}\approx 250$–300 K refer to the superparamagnetic blocking temperature $T_{B,SP}$ of the Co cores as estimated within our modified RAM framework. The exchange-bias blocking temperature for this system is discussed separately in Section 3.1, based on the observed decay of $H_{eb}$ with increasing temperature.

## 3. Discussion

### 3.1. Temperature Dependence of Coercivity and Exchange Bias

The observed non-monotonic temperature dependence of both *H_c_* and *H_eb_* with maxima in the 25–50 K range (Table 1) can be understood within the framework of spin-glass-like behavior at the Co/CoO interface, originally proposed for analogous Co/CoO systems [26]. This interpretation is particularly relevant given the core/shell morphology of our NPs [12], in which the CoO shell thickness plays a critical role in determining the exchange coupling values [26,28].

Following the model established by Thomas et al. [26], the Co/CoO interface hosts a spin-glass (SG) state with an ordering temperature below 75 K, significantly lower than the CoO Néel temperature (~150 K). The magnetic behavior is governed by competition between two coupling mechanisms: (*i*) SG-AFM coupling *J*_SG-AF_, which dominates due to the strong CoO anisotropy, and (*ii*) SG-FM coupling *J*_SG-FM_ [24,26,49,50].

At low temperatures (~5 K), strong SG-AFM coupling aligns SG moments along the CoO easy axis, minimizing energy. As temperature increases toward 75 K, thermal energy enables SG moments to reorient into metastable configurations, weakening *J*_SG-AF_ while they remain controlled by the FM Co core during magnetization reversal. This population of metastable SG moments scales with *H_c_*, explaining the observed coercivity increase up to the 25–50 K range (reaching 1.75–1.82 kOe).

Above 75 K, AFM CoO regions begin to repolarize, causing adjacent SG moments to acquire stable orientations less susceptible to FM control. This mechanism accounts for the subsequent decrease in *H_c_* from 1.46 kOe at 100 K to 0.68 kOe at 300 K. The exchange bias field follows similar trends, reaching 0.34 kOe at 25 K and decaying to ~0.014 kOe at 200 K, consistent with the progressive loss of AFM pinning.

This interpretation is supported by the observation that *H_eb_* does not vanish until temperatures approaching the exchange bias blocking temperature $T_{B,EB}$ ≈ 200–300 K, confirming that the AFM CoO shell remains magnetically active across this range [12,26]. We emphasize that this $T_{B,EB}$ is distinct from the superparamagnetic blocking temperature $T_{B,SP}$ estimated from our model in Section 2.3. The modest exchange bias values (0.1–0.34 kOe) compared to denser Co/CoO films [51] reflect the relatively sparse NPs distribution on graphene, where conglomerates that provide stronger exchange coupling are scarce. Thus, the proposed model provides a plausible semi-quantitative description of our system, albeit with the caveat that the Co/CoO/Gr system exhibits considerable heterogeneity, including size dispersion, variations in core and shell thicknesses, and the presence of conglomerates or clusters of multiple NPs. These experimental intricacies are instrumental in understanding the observed diversity of correlation functions.

It should be emphasized that the interpretation of the non-monotonic $H_{c}(T)$ and $H_{eb}(T)$ behavior in terms of spin-glass-like interfacial moments, while consistent with the well-established model for Co/CoO core/shell systems [26,27,49], remains a model-dependent hypothesis. The experimental facts, the maxima at 25–50 K and the subsequent decay, are unambiguous, but their microscopic origin cannot be uniquely determined from static magnetization measurements alone. Direct confirmation would require FC/ZFC or AC susceptibility measurements, which are beyond the scope of this work. Nevertheless, the shape of the hysteresis loops (see Figure 1 and discussion in Ref. [12]) provides indirect support for the SG interpretation. The loops exhibit an asymmetry and a shift along the field axis (exchange bias), which is characteristic of systems with uncompensated interfacial spins [48,49]. Moreover, the temperature dependence of $H_{c}$ and $H_{eb}$ (Table 1) follows the same qualitative trend as reported in the literature for Co/CoO NPs with spin-glass-like interfacial behavior [26,27]. We therefore consider the SG model to be the most consistent interpretation of our data, while recognizing that direct confirmation requires future experiments.

### 3.2. Microstructural Origins of the Correlation Functions

#### 3.2.1. Classification of Correlation Function Types

The three distinct types of CFs obtained from our analysis—non-monotonic, jump-like, and monotonic—can be associated with specific microstructural contributions to the magnetic properties of the Co/CoO NP array. The degree of NP agglomeration on graphene plays a pivotal role in determining the CF behavior, as individual NPs and their conglomerates contribute differently to the overall magnetic response. In the case of conglomerates, the packing density may be either high (closely packed NPs) or sparse, leading to distinct correlation signatures.

Non-monotonic CFs are characterized by a region of elevated amplitude with a peak at distances of (1.7–3.0)*R_NP_*, followed by a decay similar to that reported for convenient areas of FM NPs [22]. Jump-like CFs exhibit a comparable elevated amplitude region but extend to significantly larger distances of up to 7.0*R_NP_*, with abrupt changes in amplitude indicating weakly correlated regions. Monotonic CFs, in contrast, display either saturation or a gradual increase with distance *z*, indicating the presence of long-range ordering mechanisms.

It should be emphasized that a direct quantitative correlation between the reconstructed CFs and specific local morphologies (e.g., interparticle spacing distributions obtained from SEM image analysis) is beyond the scope of the present study, as it would require spatially resolved magnetic measurements combined with correlative microscopy on the same sample region. However, the assignment of CF types to microstructural configurations is strongly motivated by the following considerations. First, the SEM and HRTEM data reported in Ref. [12] explicitly show a distribution of nanoparticle diameters (centered around 100 nm) and the presence of both isolated particles and agglomerates, confirming that the system is intrinsically heterogeneous. Second, the known tendency of electrochemical deposition to occur preferentially at graphene defects and domain boundaries [52,53,54] naturally leads to spatially varying NP densities, which directly affects the magnetic correlation landscape. Third, the literature on Co/CoO core/shell systems [26,27] consistently shows that exchange bias and magnetic stability are enhanced in agglomerated configurations due to additional CoO/CoO interfaces, which is precisely the behavior reflected in the elevated amplitude regions of our non-monotonic and jump-like CFs.

Thus, while a fully quantitative microstructure-CF mapping would require future experiments combining magnetic imaging with local structural characterization, the physical plausibility of our interpretation is firmly grounded in the known properties of the system and the established physics of core/shell NPs.

#### 3.2.2. Role of NP Agglomeration and Cluster Morphology

The elevated amplitude regions observed in both non-monotonic and jump-like CFs can be attributed to the contribution of NP conglomerates, in which Co cores are positioned in proximity to one another. This configuration is consistent with the core/shell cluster assembly model described in the literature [55]. The decaying portion of the non-monotonic CF reflects the contribution of isolated, non-contacting single-core Co/CoO NPs, whereas the post-jump region in jump-like CFs characterizes weak intercluster correlations.

The enhanced CF amplitude in closely packed clusters arises from two primary factors. First, the increased fraction of CoO material enhances the correlation of anisotropy axes through strengthened exchange coupling mediated by uncompensated spins at the Co/CoO interfaces. Second, the presence of CoO/CoO interfaces, formed when NPs agglomerate during synthesis, facilitates the propagation of exchange interactions throughout the conglomerate. These interfaces arise naturally because the outer surfaces of single-core Co/CoO NPs also possess uncompensated spins, which become magnetically active upon contact between adjacent particles.

This interpretation is supported by the observation that the magnetic properties of isolated Co/CoO NPs are significantly degraded but are rapidly restored upon interparticle contact [27]. Specifically, the noticeable reduction in magnetization with increasing temperature has been attributed to the destabilization of small Co cores as the surrounding CoO matrix loses its AFM order [33]. This suggests that the primary contribution to exchange bias originates from conglomerates rather than isolated NPs. In our nanostructure, however, such conglomerates are relatively scarce, which explains why the measured *H_eb_* values are lower than those reported for denser arrays or film-like Co/CoO nanostructures [51].

A particularly intriguing feature is the increase in CF amplitude with distance observed in highly dense clusters. We propose that this behavior arises because the CoO shells effectively pin the magnetization directions of adjacent Co cores. At short interparticle distances, the correlation is comparatively weak due to competing local anisotropies; as the distance increases, the cumulative effect of random combinations of Co/CoO and interface magnetization directions leads to a net increase in correlation. In densely packed clusters, the intercore distances are substantially smaller than in isolated NP arrays, promoting stronger pinning by CoO shells (or CoO interlayers within conglomerates). This pinning enhances the effective anisotropy and facilitates ordering of the Co core magnetic moments.

#### 3.2.3. Possible Influence of Graphene Domains

Jump-like CFs are characteristic of clusters that interact weakly with one another, resulting in poor intercluster correlation of random anisotropy. Such CFs may also arise from sparse clusters, in which single-core NPs are not in intimate contact, yet the intracluster correlation remains higher than the intercluster correlation. This scenario is plausibly realized through the minimization of magnetic energy, with CoO/graphene interfaces contributing to a reduction in exchange coupling mediated by graphene via the RKKY mechanism.

Graphene is known to exhibit a domain structure [52], with domain boundaries representing defective regions characterized by reduced conductivity and diminished mechanical properties [56,57]. Importantly, defects and grain boundaries in CVD-grown graphene are well known to act as preferential nucleation sites for the electrochemical deposition of metal NPs due to their higher electrochemical reactivity [52,53,54]. It is therefore reasonable to propose that the observed jumps in the CF originate from spatially confined regions of strong anisotropy-axis correlation, arising from the self-organization of NPs into weakly interacting clusters that are separated by graphene domain boundaries. Interestingly, the characteristic distance at which these jumps occur (~70 nm) is comparable to the average graphene domain size of ~40–90 nm determined from Raman spectroscopy (see Supplementary Information, Figure S1). This suggests a plausible correlation between the spatial distribution of NP conglomerates and the underlying graphene domain structure. However, we emphasize that while the similarity of these length scales is suggestive, establishing a direct causal relationship between NP nucleation sites and the reconstructed characteristic distance would require further, spatially resolved experiments, such as correlative SEM/Raman microscopy. We therefore present this interpretation as a consistent hypothesis that is strongly motivated by established data on selective electrochemical deposition [52,53,54]. Thus, the morphology of graphene affects the density of electrochemically deposited NPs and, ultimately, the magnitude and nature of magnetic correlations.

### 3.3. Mechanisms of Long-Range Magnetic Correlations

#### 3.3.1. RKKY Interaction vs. Local Anisotropy Effect

Monotonic CFs exhibiting saturation (Figure 4c,d) demonstrate a long-range character, with coherence of the magnetic anisotropy axes extending over distances approaching one micrometer. The exchange interaction between NPs is most plausibly mediated by indirect RKKY-type coupling through the graphene layer, with an estimated magnitude not exceeding the random anisotropy contribution [16,18,21]. Nevertheless, it is difficult to attribute such extended magnetic correlations solely to indirect exchange, particularly given that the CF amplitude can even increase with distance in some cases (Figure 4e,f). This observation strongly suggests the involvement of additional mechanisms.

We propose that the long-range correlations arise from a combination of two complementary effects: (*i*) the slowly decaying RKKY interaction, which provides a distributed coupling throughout the graphene matrix, and (*ii*) the local influence of the CoO shells, which stabilize the anisotropy axes of individual Co cores through exchange bias. The CoO shell anisotropy, estimated at (2.5–2.7) × 10^7^ J/m^3^ [27,58,59], is nearly two orders of magnitude greater than typical values for polycrystalline Co NPs [45], and thus effectively pins the Co magnetization direction. The exchange bias further reinforces this stabilization. Consequently, while the RKKY interaction alone would be insufficient to maintain long-range correlations—one would indeed expect enhanced fluctuations and weakened correlations—the strong local anisotropy of each CoO shell preserves the alignment of anisotropy axes across extended distances. This dual-scale mechanism, local (shell-driven anisotropy and exchange bias per NP) and distributed (RKKY exchange through graphene), results in the observed weak decay and long-range character of the monotonic CFs.

We note that an experimental control sample consisting of bare Co NPs (without the CoO shell) on graphene would be ideal for isolating the role of the shell. However, such a sample cannot be realized because cobalt oxidizes spontaneously upon exposure to air [12,26]. While alternative systems (e.g., ferromagnetic oxide NPs) could in principle address this question, they represent a different material class and are beyond the scope of this work.

It should also be emphasized that the identification of RKKY-type exchange as the primary mechanism for the observed long-range correlations is based on a plausible interpretation rather than on direct experimental proof. Several alternative contributions could, in principle, affect the magnetic correlation landscape and should be considered.

First, dipolar interactions between NPs are always present in magnetic NP arrays and decay with distance as $E_{d}\propto 1/r^{3}$. In our system, however, the dipolar energy scale is expected to be relatively small due to the moderate saturation magnetization of the Co cores ($M_{s}∼1.1\times 10^{6}\;A/m$ [45]) and the sparse NP distribution [12]. More importantly, dipolar interactions cannot account for the monotonic increase or saturation of the CF observed in Figure 4c–f. Thus, while dipolar coupling may contribute to the overall magnetic energy, it is unlikely to be the dominant mechanism responsible for coherence of anisotropy axes over distances approaching one micrometer.

Second, cluster-mediated exchange through direct or indirect contacts between CoO shells of adjacent NPs provides a natural explanation for the enhanced CF amplitude in dense clusters (Section 3.2.2). This mechanism operates locally and is consistent with the elevated amplitude regions observed in both non-monotonic and jump-like CFs. For isolated NPs, however, separated by distances exceeding the typical cluster size, cluster-mediated exchange becomes ineffective, and a substrate-mediated coupling is required to explain the persistence of correlations.

Finally, defects and spatial variations in NP density, particularly those associated with graphene domain boundaries, introduce inhomogeneities in the local magnetic environment, as discussed in Section 3.2.3. These inhomogeneities may give rise to effective correlations that are not purely intrinsic to the NP array but reflect the underlying substrate morphology. The jump-like features in the CF are plausibly attributed to such effects.

Thus, while the RKKY mechanism provides the most consistent explanation for the long-range, monotonic CFs observed in our system, particularly given the established theoretical predictions for graphene-mediated exchange [16,18,35], we underline that our results demonstrate the existence of long-range correlations, but the exact microscopic mechanism remains a matter of interpretation. The coexistence of multiple correlation regimes within the same sample suggests that several interaction channels may operate simultaneously, with their relative importance depending on the local microstructure.

Generally speaking, the RKKY interaction in pristine graphene has been extensively studied theoretically. It has been established that, unlike conventional metals, the indirect exchange coupling in monolayer graphene exhibits a power-law decay $J(r)\propto 1/r^{3}$ [60]. Recent theoretical works have further extended this analysis to bilayer graphene, revealing that electron–phonon coupling and external fields (interlayer bias, magnetic field, and chemical potential) can significantly modulate the RKKY interaction [61,62]. These findings support our interpretation that the graphene layer mediates a slowly decaying exchange coupling between Co/CoO NPs, consistent with the long-range correlations observed in our CF analysis.

Complementary density functional theory (DFT) calculations for Co NPs supported on graphene have provided quantitative estimates of the relevant exchange interactions: FM coupling within Co metallic layers of the order of 600 K (in-plane) and 300 K (interlayer), with magnetic moments of 2.11–$1.92\;\mu_{B}$ per Co atom in the metallic layers and $0.38\;\mu_{B}$ per atom in the CoO layer [63]. Importantly, the oxidized Co layer remains ferromagnetically coupled to the underlying metallic Co, with an exchange interaction of approximately 270 K [63]. These values are in good agreement with the blocking temperatures ($T_{B,SP}\approx 250$–300 K) estimated from our model, providing independent theoretical support for the magnetic stability of the Co cores in our system.

More broadly, recent theoretical developments have highlighted the rich magnetic phase diagram of graphene-based systems. For instance, intervalley Hund’s coupling in rhombohedral graphene has been shown to favor ferromagnetic ordering [64], while the control of magnetic anisotropy through ferroelectric substrates opens new pathways for manipulating 2D magnetism [65]. Additionally, the role of disorder and local defects in stabilizing FM fluctuations in graphene has been theoretically explored [66], which is particularly relevant to our observation of graphene domain boundaries as preferential nucleation sites for NP deposition. It has also been demonstrated that the RKKY coupling between impurities on graphene becomes long-range with increasing electron-adatom interaction [67]. These studies collectively underscore the active role of graphene not merely as a passive substrate but as a versatile medium for mediating and controlling magnetic interactions in hybrid nanostructures.

#### 3.3.2. Parametric Transitions and Abrupt Changes in the Correlation Regime

The resulting dependence of the CF on the parameter *μ*, characterized by a sawtooth-like curve shown in Figure 5, indicates that the nanostructure can undergo abrupt reorganizations as *μ* varies. These sharp transitions, manifested as discontinuities in the CF at specific values of *μ_c_* and *z_c_* (Figure 4a–f), suggest that the system does not evolve continuously with changes in parameters such as NP size or density, but rather exhibits distinct, well-separated correlation regimes. The observed alternating sequence of *μ_c_* values at which CF jumps occur can be interpreted as a crossover between different regimes—for instance, transitions from strongly damped to saturated CF behavior.

We attribute these discrete rearrangements to variations in local structural configurations, such as cluster size or packing density, which directly affect the competition between local anisotropy and exchange interactions. However, it is important to emphasize that while these discontinuities are indicative of a complex energy landscape, a full interpretation in terms of critical phenomena would require establishing an appropriate order parameter and scaling behavior, which is beyond the scope of the present study. We therefore refrain from using the terminology of critical behavior and instead interpret the observed features as parametric transitions between different correlation regimes, reflecting the inherent heterogeneity of the NP array. A more detailed investigation of these abrupt changes, including their relation to local structural parameters, will be addressed in future work.

#### 3.3.3. Quantitative Parameter Estimates and Model Validation

Quantitative evaluation of the magnetic parameters within our developed framework yields values consistent with the literature reports for analogous systems. The estimated uniaxial anisotropy constant falls within the range *K* = (2.34–3.6) × 10^5^ J/m^3^, while the interfacial exchange energy per unit area is *J_eb_* = (1.8–3.0) × 10^−3^ J/m^2^, both in good agreement with previously published data (see Section 2). The blocking temperature is estimated at *T_B_* = 250–300 K, confirming the ferromagnetic state at the measurement temperatures employed. These results validate the adequacy of our approach.

Regarding the anisotropy and exchange fields, the analysis primarily establishes the ratios between *H_a_* and *H_ex_* as a function of the latter. Based on our experimental constraints and the physical considerations outlined above, we adopt the following parameter ranges for the specific sample under investigation: *H_ex_* = 0.6–1.0 kOe, *H_a_* = 4.8–6.0 kOe, and *K* = (3.0–3.6) × 10^5^ J/m^3^. This selection is motivated by several factors. First, the indirect RKKY interaction is expected to be restricted by the presence of the CoO shells, which prevent direct graphene–Co core contact. Second, the high anisotropy fields are consistent with the dominant role of CoO magnetic anisotropy, which exceeds typical Co NP values by nearly two orders of magnitude. Finally, the exchange bias effectively stabilizes the Co core magnetization.

It should be emphasized that the parameters listed in Table 2 mainly characterize the properties of individual NPs, whereas the exchange field *H_ex_* acts globally across the sample. Under these conditions, indirect exchange coupling alone is insufficient to maintain correlations between the anisotropy axes of Co NPs. In the absence of the strong local anisotropy provided by the CoO shells and exchange bias, one would anticipate enhanced magnetic fluctuations and reduced correlations. Instead, the present results demonstrate that the combination of shell-driven anisotropy and exchange bias effectively preserves long-range order by providing a local stabilizing influence for each NP. This mechanism, governed by interactions at both local and distributed scales, accounts for the weak decay and extended range of the observed correlation functions. It should be noted that in the absence of a conducting medium that couples the NPs, the magnetic correlations of NPs with core/shell morphology are not long-range [68].

### 3.4. Limitations and Role of Structural Heterogeneity

The theoretical framework employed in this work, based on the modified RAM and the Meyer integral transform, assumes an idealized 2D array of interacting NPs. However, as acknowledged throughout the discussion, the actual Co/CoO/Gr system exhibits considerable structural heterogeneity that must be considered when interpreting the extracted CFs and magnetic parameters.

First, the electrochemically deposited NPs are not monodisperse. SEM analysis reveals a distribution of diameters centered around 100 nm [12], with deviations that inevitably affect the local anisotropy and exchange coupling. Since the CFs are reconstructed from the global magnetization response M(H), the obtained C(z) functions represent an average over the entire NP ensemble, and the characteristic lengths (e.g., the peak position at $(1.7-3.0)R_{NP}$ or the jump at ${7R}_{NP}$) should be understood as effective values that may be smeared by the size dispersion.

Second, variations in the CoO shell thickness, which depend on the degree of oxidation and the local environment of each NP, directly influence the exchange-bias field $H_{eb}$ and the effective anisotropy constant K. As discussed in Section 3.1, thicker shells enhance the AFM pinning, whereas thinner shells may exhibit weaker coupling. The model parameters listed in Table 2 (e.g., $K=(2.34-3.6)\times 10^{5}\;J/m^{3}$ and $J_{eb}=(1.8-3.0)\times 10^{-3}\;J/m^{2}$) represent effective values averaged over the distribution of shell thickness, and the actual local values may deviate substantially from these averages.

Third, the degree of NP agglomeration plays a crucial role in determining the type of CF (monotonic, non-monotonic, or jump-like), as discussed in Section 3.2. Dense clusters exhibit enhanced correlations due to CoO/CoO interfaces and interparticle exchange, whereas isolated NPs contribute to the decaying tail of the CF. The coexistence of these different configurations within the same sample implies that the reconstructed C(z) is a weighted superposition of contributions from isolated particles, small clusters, and larger conglomerates. This heterogeneity also explains why the exchange-bias values used here ($H_{eb}\approx 0.1–0.34$ kOe) are lower than those reported for denser Co/CoO films, where cluster contributions dominate.

Finally, the role of graphene domain boundaries, which act as preferential nucleation sites for electrochemical deposition [52,53,54], introduces spatial variations in the local NP density. The observed jump-like features in the CF at distances of ∼70 nm are tentatively attributed to these domain boundaries, but we acknowledge that a more direct confirmation would require spatially resolved measurements, such as correlative SEM/Raman microscopy.

In summary, while the modified 2D LAMS framework provides a powerful tool for extracting the main micromagnetic parameters from global magnetization data, the reconstructed CFs and fitted parameters should be interpreted as effective, ensemble-averaged quantities. The structural heterogeneity inherent to the Co/CoO/Gr system does not invalidate the main conclusions regarding the existence of long-range magnetic correlations and the distinct CF regimes, but it does introduce uncertainties that should be taken into account when comparing the results with those of more homogeneous systems. Future work combining magnetic measurements with local structural characterization at the single-particle level would be valuable for refining the model and establishing a more direct correspondence between microstructure and magnetic correlations.

## 4. Conclusions

In this work, we have systematically investigated the low-temperature magnetic behavior of Co/CoO core–shell NP arrays electrochemically deposited on graphene, with a focus on the 25–50 K temperature range where the coercivity and exchange bias reach their maximum values. While the experimental data (synthesis, structure, and basic magnetization) were reported previously in Ref. [12], the present contribution lies in the application of a modified 2D LAMS framework to extract, for the first time, the real-space correlation functions and a comprehensive set of micromagnetic parameters for this system. By applying this framework, specifically adapted for 2D magnetic systems via the Meyer integral transform, we have reconstructed the real-space correlation functions C(z) of the magnetic anisotropy axes and extracted key micromagnetic parameters, including the random anisotropy field *H_a_*, exchange field *H_ex_*, uniaxial anisotropy constant *K*, interfacial exchange energy *J_eb_*, and blocking temperature *T_B,SP_*. These parameters should be interpreted as effective, ensemble-averaged values, reflecting the inherent structural heterogeneity of the NP array. Our analysis reveals three distinct types of correlation functions—monotonic, non-monotonic, and jump-like—each reflecting different microstructural configurations and interaction regimes within the NP array. Non-monotonic CFs, characterized by a peak at (1.7–3.0)*R_NP_* followed by decay, are associated with isolated NPs and weakly interacting clusters. Jump-like CFs, exhibiting abrupt amplitude changes at distances up to 7*R_NP_*, correspond to sparse clusters separated by graphene domain boundaries, with the characteristic jump distance (~70 nm) closely matching the graphene grain size determined from Raman spectroscopy. Monotonic CFs with saturation or increasing amplitude demonstrate long-range correlations extending over nearly one micrometer, a striking and, to our knowledge, previously unreported finding for this class of hybrid nanomaterials. These experimental findings are consistent with recent theoretical predictions of long-range RKKY-mediated exchange in graphene [16,18,67] and DFT calculations of ferromagnetic coupling in Co/CoO NPs on graphene [63], confirming the active role of graphene as a mediator of magnetic correlations.

The origin of these long-range correlations is attributed to a dual-scale mechanism: (*i*) indirect RKKY-type exchange through the graphene layer, which provides a distributed coupling, and (*ii*) the strong local anisotropy of the CoO shells (approximately 10^7^ J/m^3^), which pins the magnetization direction of individual Co cores via exchange bias. This combination effectively overcomes the rapid decay typically associated with random anisotropy models, enabling coherence of anisotropy axes across extended distances. The estimated micromagnetic parameters, such as *K* = (2.34–3.6) × 10^5^ J/m^3^, *J_eb_* = (1.8–3.0) × 10^−3^ J/m^2^, and the superparamagnetic blocking temperature *T_B,SP_* = 250–300 K, are in excellent agreement with the literature values, validating the adequacy of our theoretical approach. Furthermore, the observed dependence of the correlation function on the critical parameter *µ* reveals evidence of critical phenomena and system self-organization, with sharp transitions at *µ_c_* suggesting discrete structural or magnetic reorganizations. These findings underscore the rich and diverse physics governing magnetic interactions in 2D hybrid systems, where nanoscale order can be precisely mapped and, potentially, controlled on the micrometer scale. The non-monotonic temperature dependence of *H_c_* and *H_eb_*, with a maximum in the 25–50 K range, is convincingly explained by the reorientation of spin-glass moments at the Co/CoO interfaces. This interpretation highlights the pivotal role of uncompensated interfacial spins and the competition between AFM and FM coupling, providing a comprehensive picture of the magnetic behavior in core–shell nanoparticles.

The results obtained demonstrate that graphene serves not merely as a passive substrate but as an active mediator of magnetic interactions, capable of sustaining long-range correlations through its unique electronic structure. This finding is of fundamental interest for the field of 2D magnetism and establishes a quantitative framework for understanding magnetic order in hybrid graphene-based systems. From a practical perspective, the observation of such extended magnetic correlations suggests that graphene-decorated magnetic NPs could potentially be relevant for future spintronic applications, provided that efficient spin transport can be achieved and that the magnetic stability can be maintained at relatively high temperatures. However, we emphasize that the present study is based on static magnetic measurements at low temperatures, and the demonstration of device functionality, such as spin filtering or magnetoresistance switching, requires additional research. The extracted micromagnetic parameters presented here thus serve as a foundation for future investigations, which should combine magnetic correlation analysis with transport measurements and room-temperature studies to fully assess the spintronic potential of these hybrid systems. Additionally, the phenomena suggested by the sawtooth-like dependence of *C*(*µ*) merit a dedicated theoretical and experimental investigation. While the proposed dual-scale mechanism provides the most consistent explanation for the observed long-range correlations, we acknowledge that direct experimental confirmation of RKKY-mediated exchange in this system requires further investigation, for instance through spatially resolved magnetic imaging or transport measurements.

## Figures and Tables

**Figure 1:**
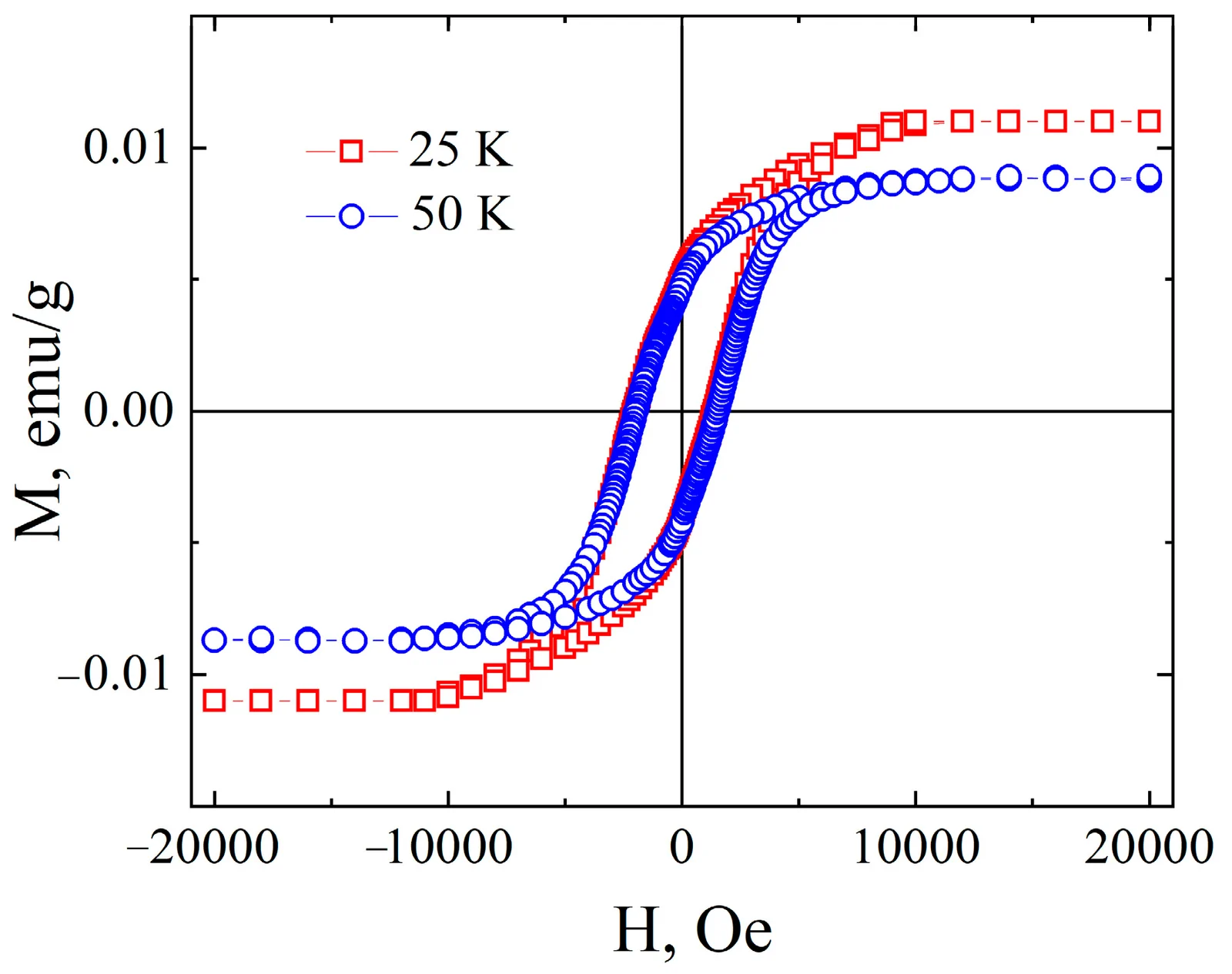
*M*(*H*) dependencies measured at *T* = 25 K and 50 K using the field-cooled protocol (*H* = +10 kOe).

**Figure 2:**
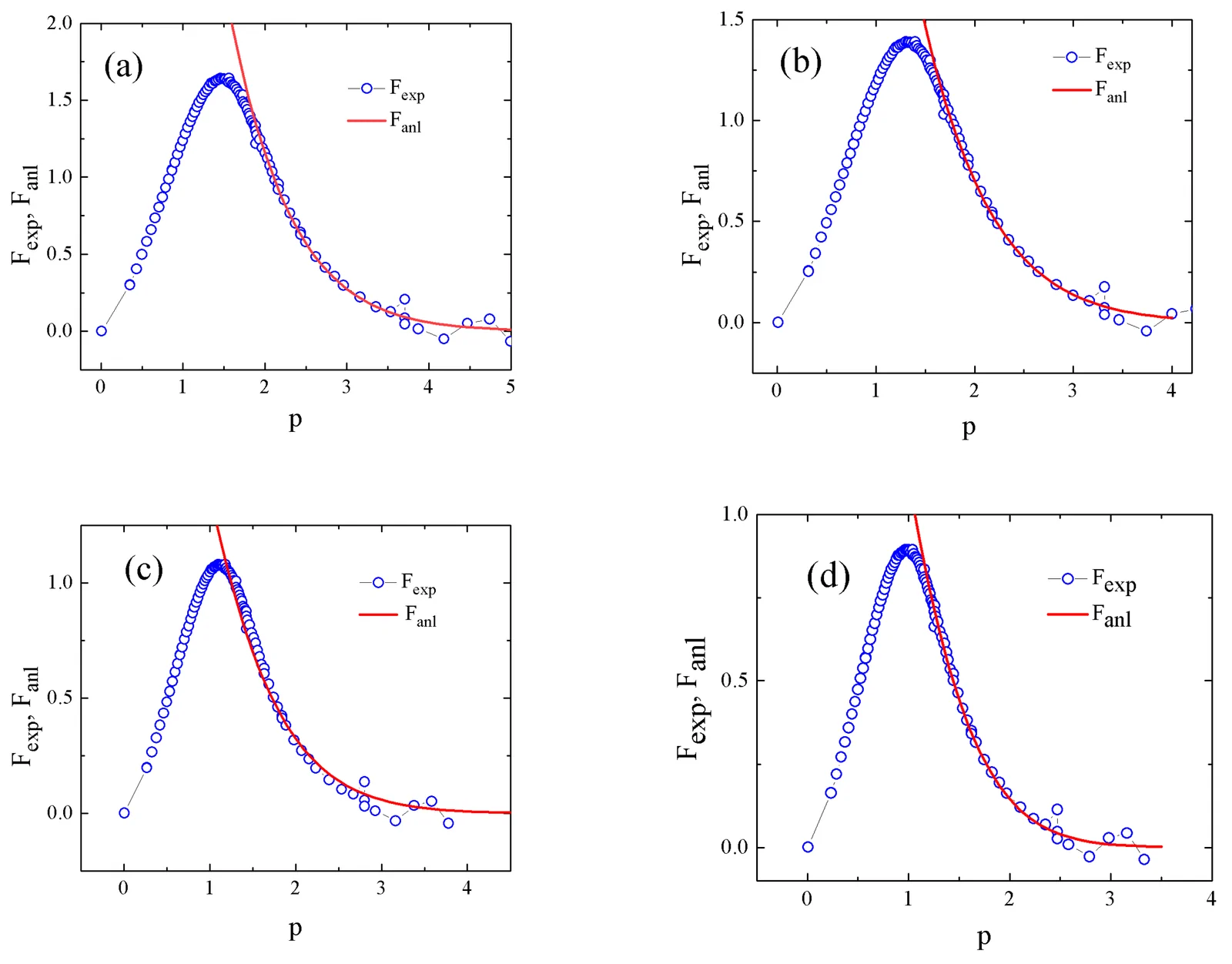
Experimental *F_exp_*(*p*) (blue circles) and analytical *F_anl_*(*p*) curves (red solid lines) at *T* = 50 K and *a* = 1.0, plotted for different exchange fields *H_ex_*: (**a**) 800 Oe, (**b**) 1000 Oe, (**c**) 1400 Oe, and (**d**) 1800 Oe. The experimental data are calculated using Equation (2), and the analytical curves are obtained from Equation (3) with *µ* ranging from –0.8 to –0.6.

**Figure 3:**
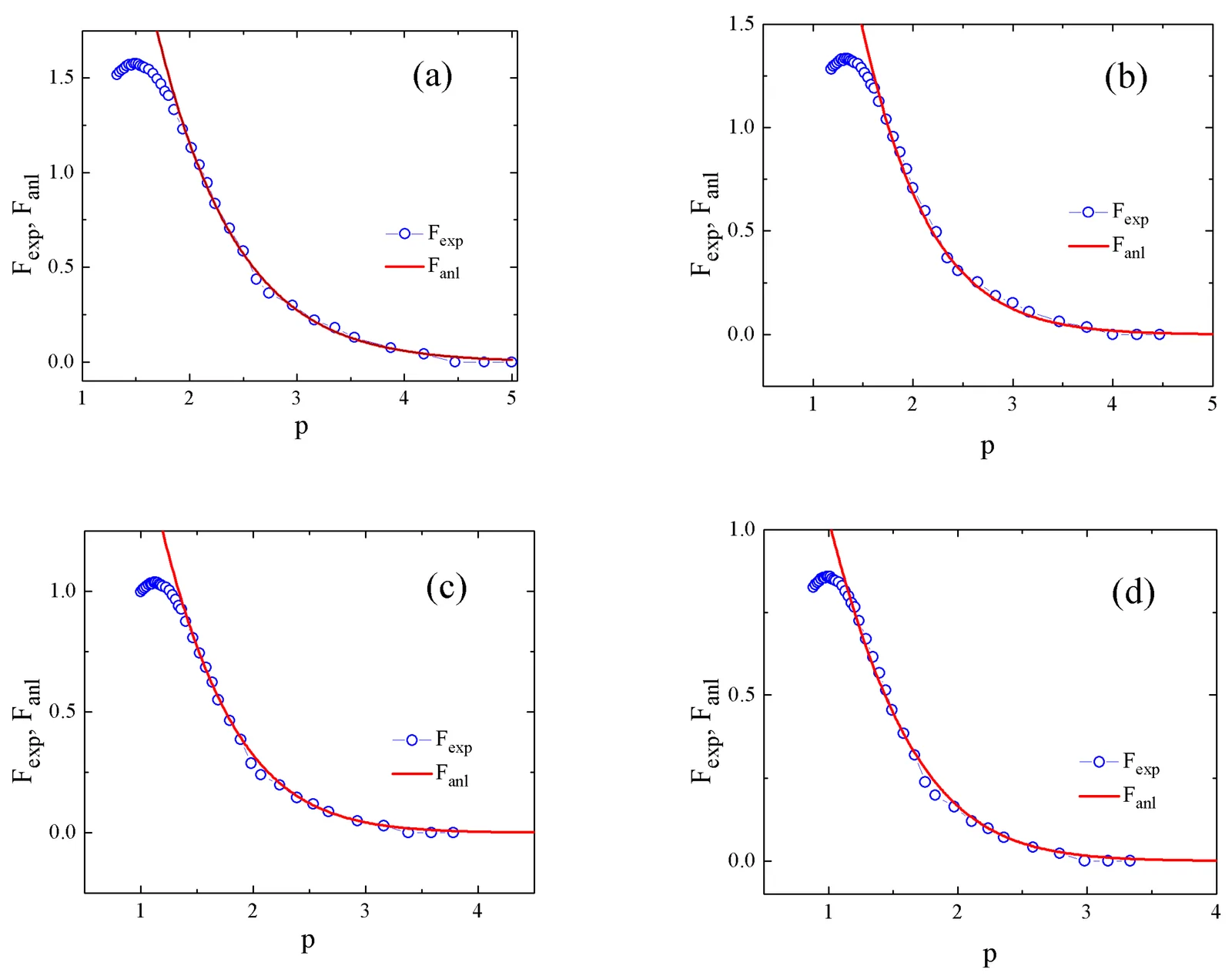
Experimental *F_exp_*(*p*) (blue symbols with lines) and analytical *F_anl_*(*p*) curves (red solid lines) at *T* = 25 K and *a* = 1.0, plotted for different exchange fields *H_ex_*: (**a**) 800 Oe, (**b**) 1000 Oe, (**c**) 1400 Oe, and (**d**) 1800 Oe. The experimental data are calculated using Equation (2), and the analytical curves are obtained from Equation (3) with *µ* ranging from –0.8 to –0.6.

**Figure 4:**
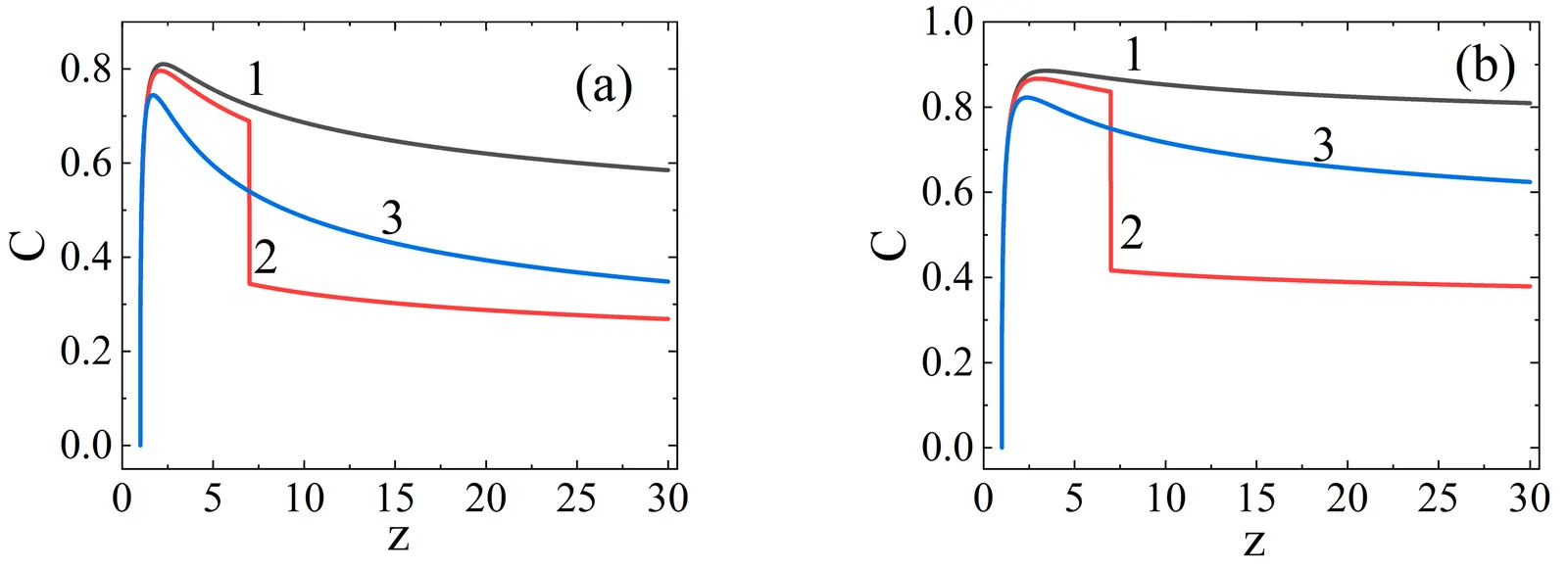

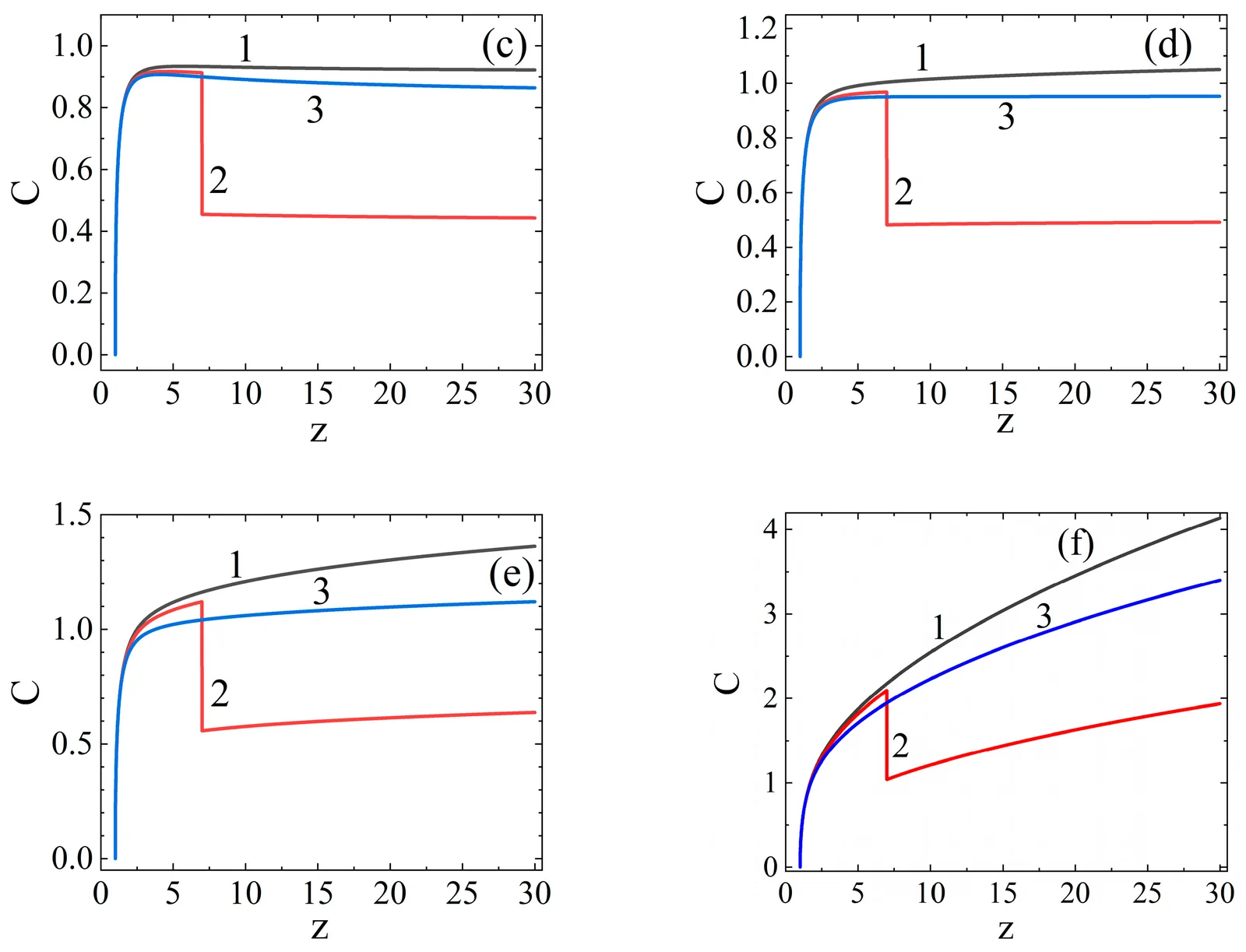
Real-space magnetic anisotropy correlation functions *C*(*z*) calculated using the inverse Meyer transform for *a* = 1.0 and different *µ*: (**a**) −0.68 (1), −0.667 (2), −0.6 (3); (**b**) −0.73 (1), −0.72 (2), −0.69 (3); (**c**) −0.75 (1), −0.744 (2), −0.74 (3); (**d**) −0.77 (1), −0.76 (2), −0.755 (3); (**e**) −0.81 (1), −0.8 (2), −0.78 (3); and (**f**) −0.98 (1), −0.97 (2), −0.95 (3).

**Figure 5:**
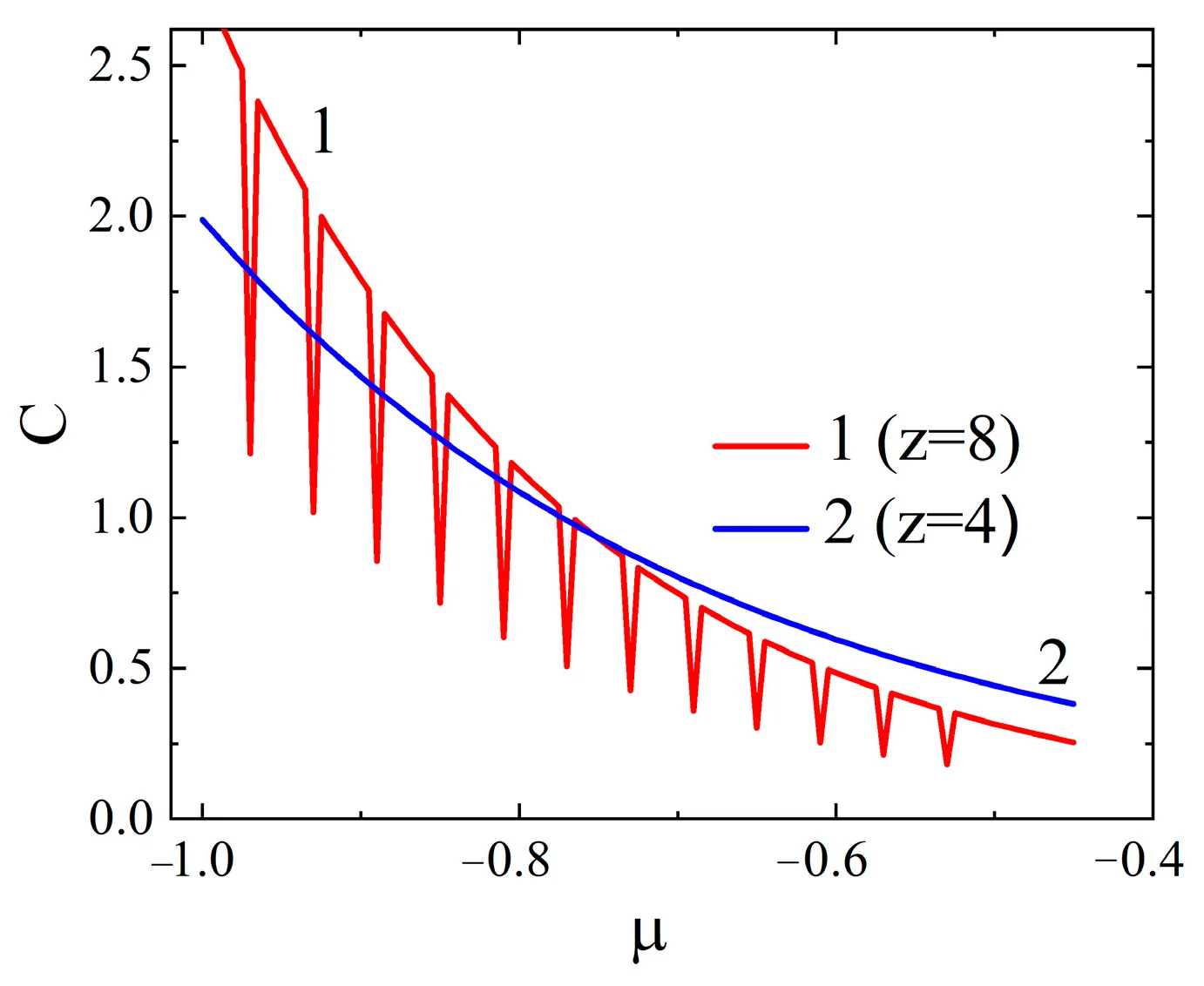
The correlation function *C* plotted as a function of the parameter *µ* for *z* = 8 > *z_c_* (curve 1) and *z* = 4 < *z_c_* (curve 2), where *z_c_* = 7.0 defines the critical spatial coordinate at which the discontinuous amplitude jump occurs.

**Figure 6:**
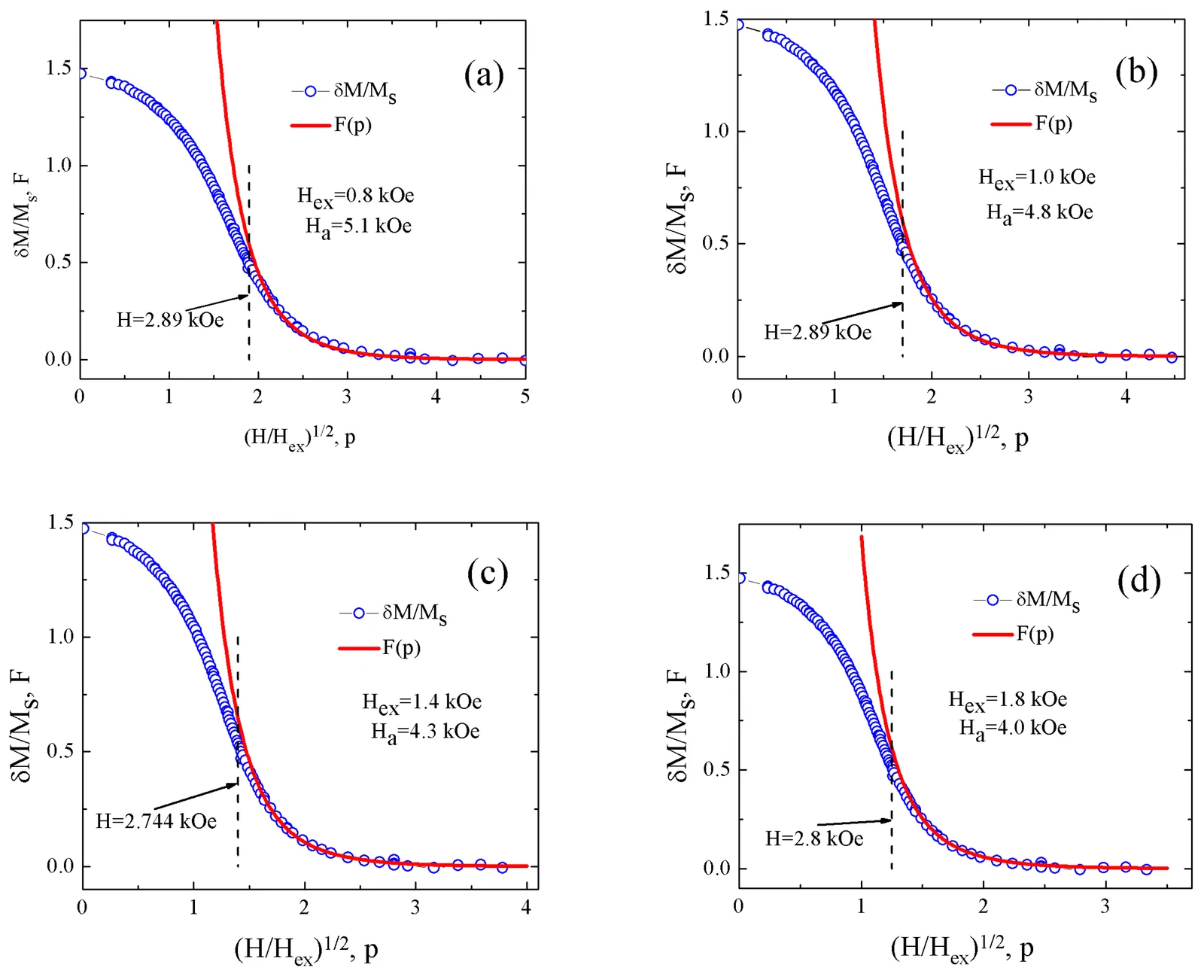
Experimental (symbols) *δM*(*H*)/*M_S_* and calculated (solid lines) *F*(*p*) dependencies for Co/CoO/Gr sample at *T* = 50 K for various *H_ex_*: (**a**) 0.8 kOe, (**b**) 1.0 kOe, (**c**) 1.4 kOe, and (**d**) 1.8 kOe. The vertical dashed lines indicate the fields above which the LAMS is valid.

**Figure 7:**
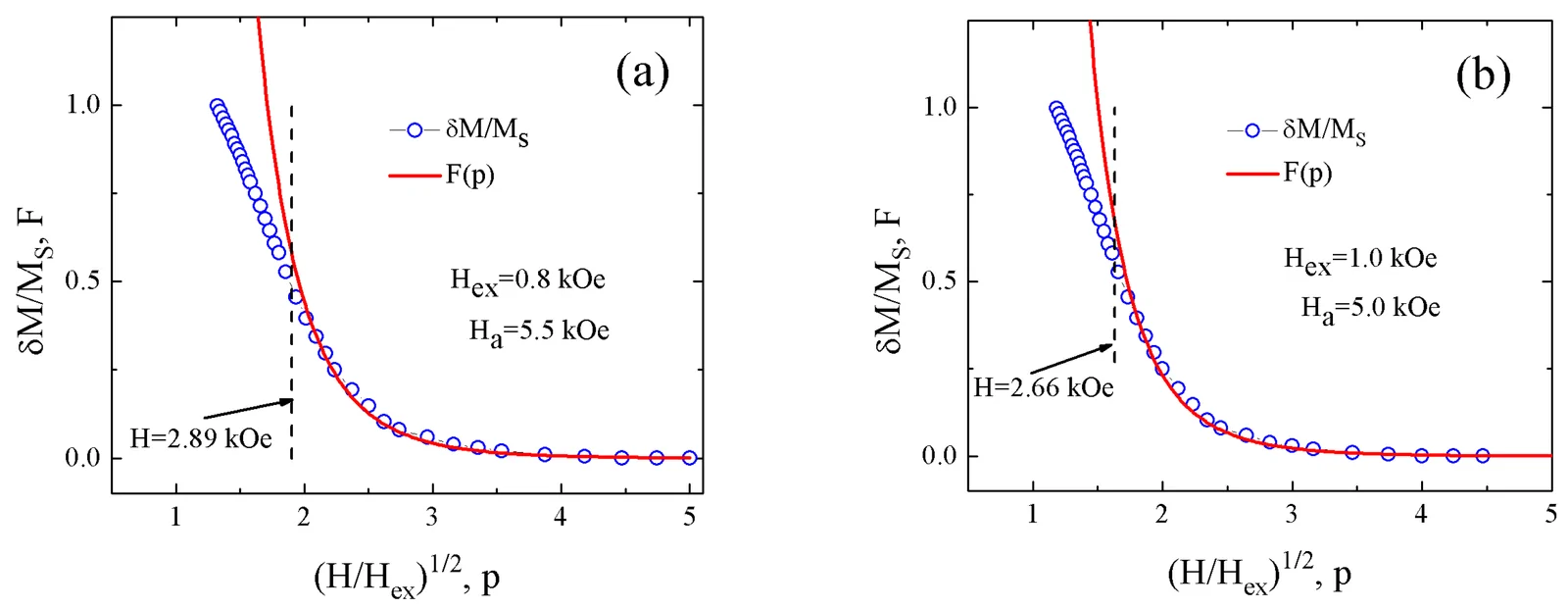

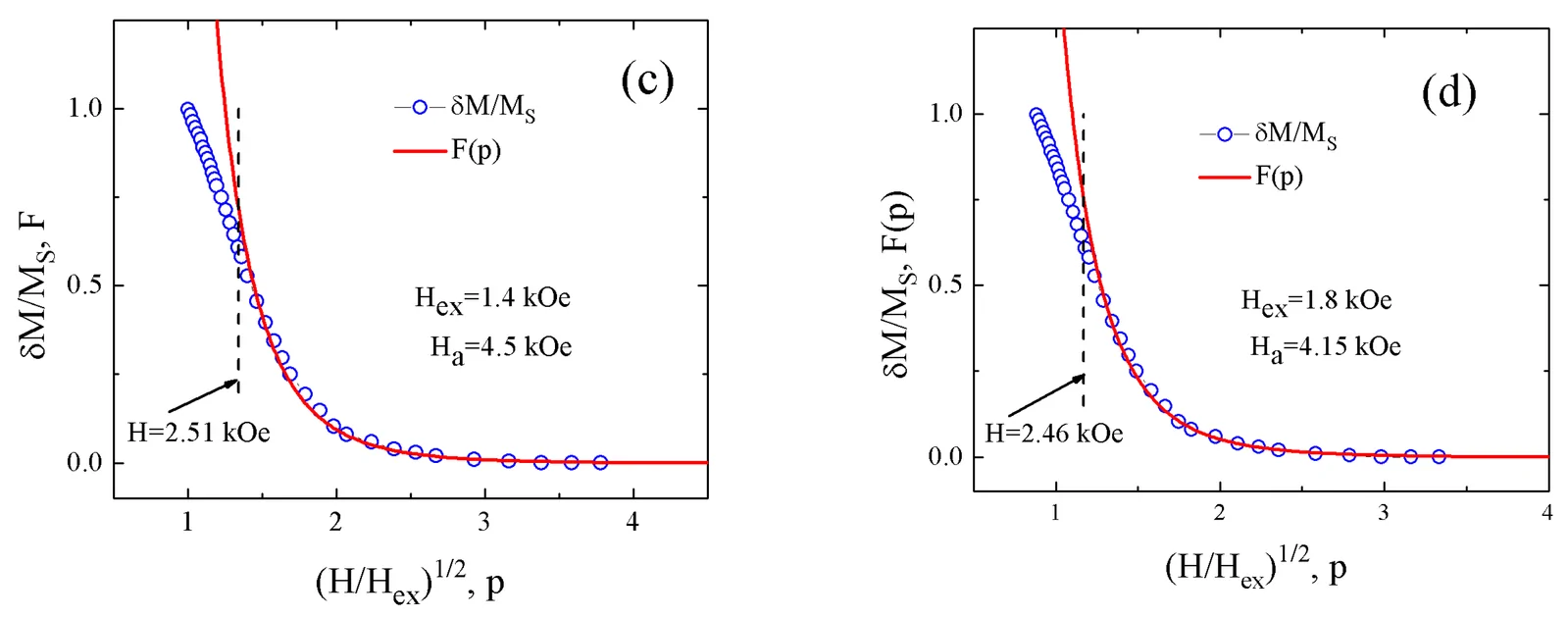
Experimental (symbols) *δM*(*H*)/*M_S_* and calculated (solid lines) *F*(*p*) dependencies for Co/CoO/Gr sample at *T* = 25 K for various *H_ex_*: (**a**) 0.8 kOe, (**b**) 1.0 kOe, (**c**) 1.4 kOe, and (**d**) 1.8 kOe. The vertical dashed lines indicate the fields above which the LAMS is valid.

**Figure 8:**
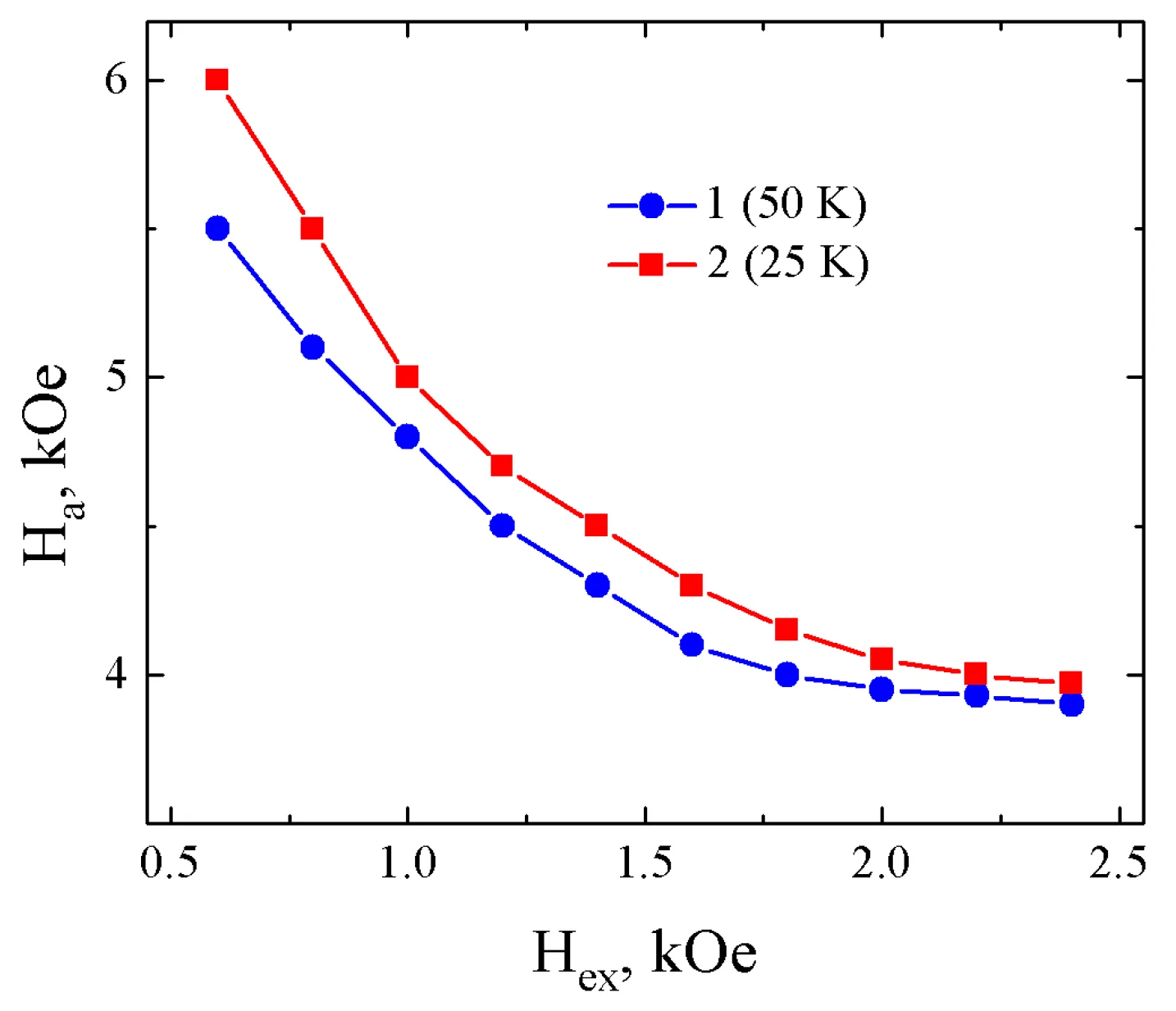
The dependence of the anisotropy field $H_{a}$ on the exchange field *H_ex_* at *T* = 50 K (curve 1) and *T* = 25 K (curve 2).

**Figure 9:**
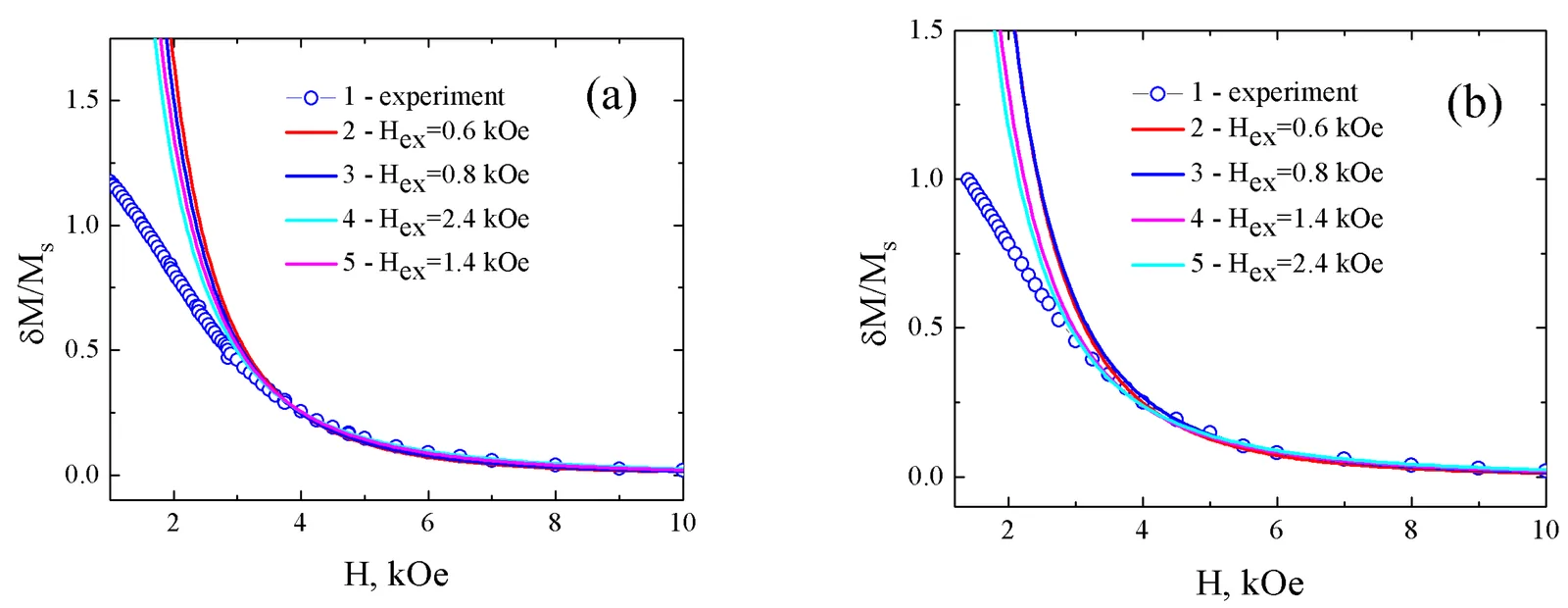
Experimental (symbols) and calculated (solid lines) *δM*(*H*)/*M_S_* dependencies for Co/CoO/Gr sample. (**a**) *T* = 50 K; (**b**) *T* = 25 K.

**Table 1:** Magnetic parameters of the Co/CoO NPs on graphene as obtained from the *M*(*H*) measurements.

*T*, K	*H_c_*, kOe	*H_eb_*, kOe	*M_S_*, emu/g
4	0.549	0.1565	0.0164
25	1.820	0.3409	0.0110
50	1.750	0.2116	0.0091
100	1.458	0.0522	0.0089
200	0.970	0.0139	0.0091
300	0.682	0.0079	0.0091

**Table 2:** Magnetic parameters of the Co/CoO NPs array on graphene *.

*H_c_*, kOe	*T_B_*, K	*A*, ×10^−11^ J/m	*H_ex_*, kOe	*H_a_*, kOe	*K*, ×10^5^ J/m^3^	*R_NP_*, nm	*J_eb_*, mJ/m^2^
*T* = 50 K							
1.75	250–295	1.2–2.0	0.6–2.4	3.9–5.5	2.34–3.3	8–12	1.8–2.9
*T* = 25 K							
1.82	250–300	1.2–2.0	0.6–2.4	3.97–6.0	2.4–3.6	8–12	1.9–3.0

* Note: The parameter ranges listed in this Table represent effective values obtained from the ensemble-averaged magnetization data. Local variations due to NP size dispersion, shell thickness, and cluster morphology may deviate from these averages.

## Data Availability

The original contributions presented in this study are included in the article. Further inquiries can be directed to the corresponding author.

## Supplementary Materials

The following supporting information can be downloaded at https://www.mdpi.com/article/10.3390/nano16191201/s1, References [69,70] are cited in the supplementary materials. Figure S1: Distribution of domain sizes of graphene as obtained from the Raman spectra. Inset: Example of a single Raman spectrum of graphene; Figure S2: (a) Comparison of experimental and calculated dependences of δ*M*(*p*)/*M_s_*, Figure 6, at *T* = 50 K and various *H_ex_*: (1) 1.0 kOe, (2) 1.4 kOe, (3) 2.4 kOe. The RMSE values on curves 1 and 2 that exceed 0.1 occur in the region of significant discrepancy between the experimental and calculated dependences of δ*M*(*p*)/*M_s_*; (b) Comparison of experimental and calculated dependences of δ*M*(*p*)/*M_s_* (Figure 7) at *T* = 25 K and various *H*ex values: (1) 2.4 kOe, (2) 2.0 kOe, (3) 1.2 kOe, (4) 1.0 kOe. The RMSE values on curves 3 and 4 that exceed 0.1 occur in the region of significant discrepancy between the experimental and calculated dependences of δ*M*(*p*)/*M_s_*.
