# Supplementary material for: Magnetic Correlations in Co/CoO Core/Shell Nanoparticles Decorated on Graphene: Insights from a Modified 2D Law of Approaching Magnetic Saturation
Source: Nanomaterials (Basel). 2026 Sep 23;16(19):1201. doi: 10.3390/nano16191201 (PMC13667030; doi:10.3390/nano16191201)
Supplement: Supplementary file 1 [file nanomaterials-16-01201-s001.zip › nanomaterials-4540294-supplementary.pdf]

Magnetic Correlations in Co/CoO Core/Shell Nanoparticles Decorated on Graphene: Insights from a Modified 2D Law of Approaching Magnetic Saturation

E.S. Nazarenka[1], A.L. Danilyuk[1], N.G. Kovalchuk[2], A.O. Konakov[3], J.A. Fedotova[4], S.L. Prischepa[5]

[1]Department of Micro- and Nanoelectronics, Belarusian State University of Informatics and Radioelectronics, 220013 Minsk, Belarus; katyanaz2001@gmail.com (E.S.N.); danilyuk@bsuir.by (A.L.D.)

[2]Laboratory "Integrated Micro- and Nanosystems", Research and Development Department, Belarusian State University of Informatics and Radioelectronics, 220013 Minsk, Belarus; n.kovalchuk@bsuir.by

3 Research Institute for Physical Chemical Problems, Belarusian State University, 220030, Minsk, Belarus; artjom7777@gmail.com

[4] Institute for Nuclear Problems, Belarusian State University, 220030, Minsk, Belarus; julia@hep.by

[5] Department of Information Security, Belarusian State University of Informatics and Radioelectronics, 220013 Minsk, Belarus; prischepa@bsuir.by

The Tuinstra-Koenig equation was used to calculate the domain size L_a of few-layer graphene [69]:

$$L_a(nm) = (2.4 \times 10^{-10})\lambda_L^4 \left(\frac{I_G}{I_D}\right) \quad (1)$$

Where λ_L is the wavelength of the laser, I_G and I_D are amplitudes of the G and D lines on the Raman spectra of graphene.

This equation is valid for graphene domain sizes larger than the phonon coherence length, which is found to be equal to ~32 nm [70]. For a Raman with a 473 nm laser, Eq. (1) yields:

$$L_a(nm) \approx 12.02 \left(\frac{I_G}{I_D}\right)$$

Ratio I_G/I_D for graphene before decoration by cobalt nanoparticles was calculated from the Raman spectra. In total, 100 spectra were recorded within the area 10μm×10μm. The example of a single spectrum is shown in the inset to Figure SI-1. In the main panel of this Figure, the distribution of the domain sizes is plotted. We found that most of the domains have average sizes in the range between 50 and 100 nm.

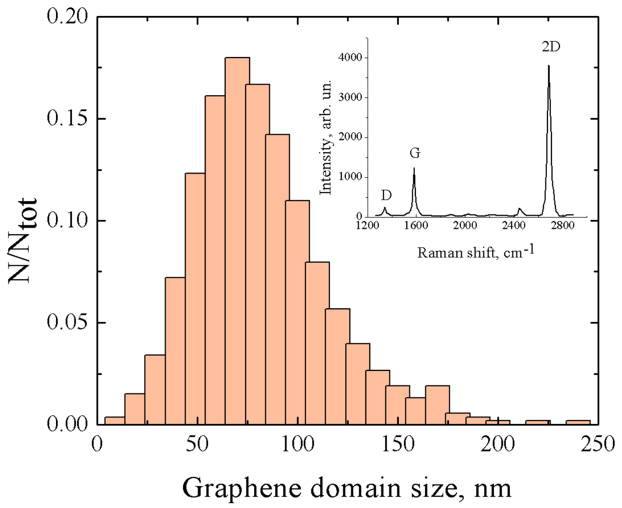

Figure S1. Distribution of domain sizes of graphene as obtained from the Raman spectra. Inset: Example of a single Raman spectrum of graphene.

The root-mean-square error (RMSE) was calculated according to the expression $RMSE = \sqrt{\frac{1}{N}\left(F_{exp} - F_{anl}\right)^2}$. The results are presented in Figures SI-2a (T = 50 K) and SI-2b (T = 25 K).

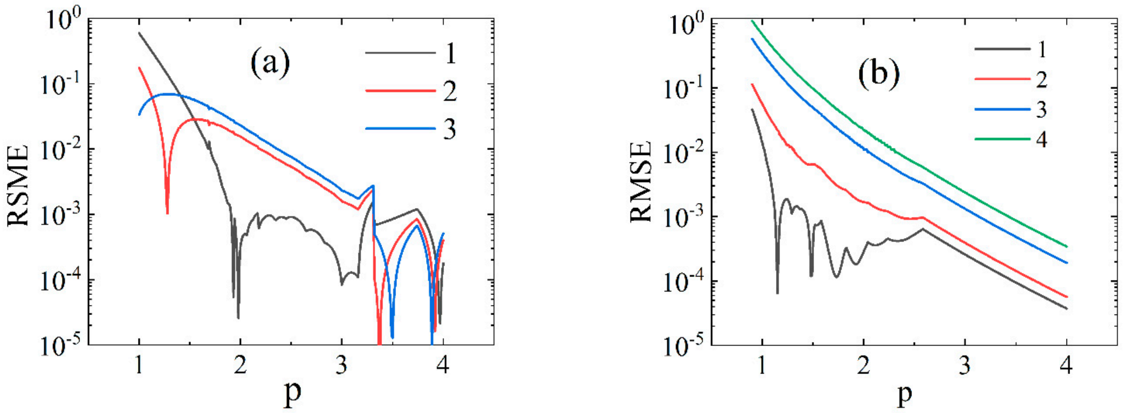

Figure S2. (a) Comparison of experimental and calculated dependences of $\delta M(p)/M_s$, Fig. 6, at T = 50 K and various H_{ex}: (1) 1.0 kOe, (2) 1.4 kOe, (3) 2.4 kOe. The RMSE values on curves 1 and 2 that exceed 0.1 occur in the region of significant discrepancy between the experimental and calculated dependences of $\delta M(p)/M_s$; (b) Comparison of experimental and calculated dependences of $\delta M(p)/M_s$ (Fig. 7) at T = 25 K and various H_{ex} values: (1) 2.4 kOe, (2) 2.0 kOe, (3) 1.2 kOe, (4) 1.0 kOe. The RMSE values on curves 3 and 4 that exceed 0.1 occur in the region of significant discrepancy between the experimental and calculated dependences of $\delta M(p)/M_s$.

[1] Pimenta, M.A.; Dresselhaus, G.; Dresselhaus, M.S.; Cançado, L.G.; Jorio, A.; Saito, R. Studying disorder in graphite-based systems by Raman spectroscopy. Phys. Chem. Chem. Phys. 2007, 9, 1127–1290. https://doi.org/10.1039/b613962k.

[2] Ribeiro-Soares, J.; Olivero, M.E.; Garin, C.; David, M.V.; Martins, L.G.P.; Almeida, C.A.; Martins-Ferreira, E.H.; Takai, K.; Enoki, T.; Magalhães-Paniago, R.M.; et al. Structural analysis of polycrystalline graphene systems by Raman spectroscopy. Carbon 2015, 95, 646–652. https://doi.org/10.1016/j.carbon.2015.08.020.
